# EEG signature in psychiatrist’s subjective experience: a pilot study

**DOI:** 10.3389/fpsyt.2026.1933800

**Published:** 2026-10-05

**Authors:** Giovanni Videtta, Elena Bondi, Chiara Colli, Gabriele Enrico, Luca Sperti, Mauro Pallagrosi, Laura Fonzi, Ylenia Barone, Giandomenico Schiena, Eleonora Maggioni, Angelo Picardi, Paolo Brambilla

**Affiliations:** 1 Department of Neurosciences and Mental Health, Fondazione IRCCS Ca’ Granda, Ospedale Maggiore Policlinico, Milan, Italy; 2 Department of Pathophysiology and Transplantation, University of Milan, Milan, Italy; 3 Rome 1 Local Health Unit, Community Service for Prevention and Early Intervention in Mental Health, Rome, Italy; 4 Reverie Psychiatric Residential Facility, Rome, Italy; 5 Department of Electronics Information and Bioengineering, Politecnico di Milano, Milan, Italy; 6 Centre for Behavioural Sciences and Mental Health, Italian National Institute of Health, Rome, Italy

**Keywords:** electroencephalography, intersubjectivity, neurobiological correlates, phenomenology, subjective experience

## Abstract

**Introduction:**

The Assessment of Clinician’s Subjective Experience (ACSE) is a self-report questionnaire developed to evaluate clinicians’ subjective experiences during patient interactions. While its psychometric properties and clinical validity have been supported, its neurobiological correlates have not yet been investigated.

**Methods:**

In this pilot study, two clinicians and ten patients participated in a three-phase protocol comprising: (1) a first clinical interview, (2) ACSE administration, and (3) listening to the audio recording of the first clinical interview. EEG activity was recorded from clinicians during both the first clinical interview and questionnaire administration. Neural data were analyzed in terms of relative power spectral density, node strength, and global efficiency computed from imaginary coherence.

**Results:**

Clinicians were psychiatrists, one male and one female. Seven patients were diagnosed with mixed anxiety and depressive disorder, and three with unspecified anxiety disorders. ACSE scores were highest for the Difficulty in attunement and Engagement dimensions, consistent with previous literature. EEG findings revealed dimension-specific and topographically distinct oscillatory patterns for only two ACSE dimensions: Difficulty in Attunement and Engagement. More limited evidence emerged for Disconfirmation. Functional connectivity analyses indicated different network profiles, characterized by differences in global efficiency patterns. Correlational analyses further identified significant associations between ACSE dimensions and EEG-derived features.

**Discussion:**

These preliminary findings provide initial evidence that clinicians’ subjective experiences during clinical encounters could be associated with measurable neural signatures, offering a novel perspective on the neurobiological basis of intersubjective processes in clinical settings.

## 1 Introduction

Phenomenological psychiatry has long emphasized the crucial role of the clinician’s lived perception of the patient in the diagnostic process (1), arguing that exclusively objective or externalizing approaches are insufficient to capture the qualitative complexity of psychiatric phenomena (2, 3). More recent theoretical frameworks conceptualize psychiatric diagnosis as a dialogical and intersubjective process, in which the clinician’s perceptual, emotional, and cognitive responses actively contribute to the identification of the structural features of mental disorders (4, 5).

Over the past decade, renewed interest has emerged in empirically investigating the phenomenological processes that shape clinical encounters, driven by the need for standardized instruments capable of capturing and quantifying clinicians’ intersubjective experiences. Within this context, the Assessment of Clinician’s Subjective Experience (ACSE) was developed as a structured self-report questionnaire designed to assess clinicians’ experiential and affective states during clinical encounters (6). The ACSE is completed immediately after the first clinical interview, and assesses five core dimensions of subjective experience Tension, Difficulty in Attunement, Engagement, Disconfirmation, and Impotence. By operationalizing constructs grounded in phenomenological psychopathology, the ACSE enables systematic quantification of relational phenomena that have traditionally been explored only qualitatively (7).

Empirical studies using the ACSE have demonstrated that clinicians’ subjective experiences can be measured reliably and carry substantial clinical and diagnostic relevance. ACSE scores vary systematically across diagnostic categories and levels of symptom severity, indicating that clinicians’ experiential responses are strongly related to patients’ clinical conditions (6). Psychotic disorders, particularly schizophrenia, are associated with elevated levels of Difficulty in Attunement, Tension, and Impotence, distinguishing schizophrenia from other psychotic conditions (8). In contrast, mood and anxiety disorders tend to be associated with lower scores across multiple ACSE dimensions, while personality disorders show distinct experiential profiles with high Disconfirmation and low Engagement (9). Furthermore, ACSE dimensions show meaningful correlations with symptom domains assessed by established clinical instruments, including the Brief Psychiatric Rating Scale, and symptom severity has been shown to be associated with clinicians’ subjective responses across several ACSE dimensions (10). These experiential patterns are also closely linked to therapeutic collaboration and countertransference processes, with more negative subjective experiences associated with poorer therapeutic alliance (11). Importantly, the ACSE has demonstrated robust internal and external validity across different patient age groups, including adolescents (12), cultural contexts (13), and clinician characteristics, including gender (14).

To date, however, the validation and application of the ACSE have been confined to the clinical and phenomenological levels, without direct investigation of the neurobiological processes that may underlie clinicians’ subjective experiences. In this regard, an expanding body of research has identified electroencephalography (EEG) as a promising approach for examining the neurobiological correlates of intersubjective processes (15). EEG is a non-invasive functional technique that records brain electrical activity generated by postsynaptic neuronal potentials (16) and offers high temporal resolution, enabling the investigation of neural dynamics as they unfold in real time (17). EEG is particularly well suited to investigate the neurobiological basis of clinicians’ subjective experiences because it provides an objective measure of neural activity with millisecond temporal resolution, enabling the detection of rapid changes in brain dynamics occurring during live interpersonal interactions. In this respect, EEG complements the ACSE by capturing the neurophysiological processes that accompany subjective experience before they are retrospectively reported by the clinician.

Recent EEG research has increasingly focused on clinician-patient interactions, reflecting growing recognition of the reciprocal emotional, cognitive, and physiological influences inherent in interpersonal exchanges (18). In particular, hyperscanning paradigms, allowing simultaneous recording of neural activity from interacting individuals, have revealed patterns of inter-brain synchrony linked to empathy, affective attunement, and social cognition (19). EEG studies have further demonstrated that clinicians’ neural responses may index implicit emotional reactions and biases (20), with specific neural signatures associated with emotional engagement, distress, and empathic resonance (21–23). Notably, higher levels of neural synchrony between clinician and patient have been associated with stronger therapeutic alliance and emotional alignment, underscoring the relevance of neurobiological processes in effective clinical relationships (24–26).

Within this theoretical and empirical framework, investigating the neurobiological underpinnings of the clinician’s subjective experience may yield important insights into the mechanisms of empathy, attunement, and emotional resonance that shape clinical interactions, with potential implications for diagnostic accuracy and therapeutic efficacy. Accordingly, the present pilot study aims to explore the neurobiological correlates of clinicians’ subjective experiences during patient interactions by integrating EEG measures with the ACSE. Although specific predictions regarding the association between individual ACSE dimensions and EEG frequency bands could not be established due to the lack of previous evidence, we expected that ACSE dimensions would be associated with measurable differences in oscillatory activity and functional connectivity, reflecting the involvement of neural mechanisms underlying clinicians’ subjective experiences. By combining neurobiological and self-report approaches, this study seeks to bridge clinical phenomenology and its underlying neural mechanisms, offering a novel framework for understanding the role of the clinician’s subjective experience in mental health care.

## 2 Materials and methods

### 2.1 Participants

Two clinicians and ten patients were recruited at the Psychiatry Unit of Fondazione IRCCS Ca’ Granda Ospedale Maggiore Policlinico, Milan, Italy. Each clinician conducted first clinical interviews with five patients. Inclusion criteria for clinicians were: (i) Italian as a mother tongue and (ii) absence of contraindications to EEG recording, including a history of epileptic seizures, neurological lesions, or intracranial devices. No specific inclusion criteria were applied to patients. Psychiatric diagnoses were established according to the International Statistical Classification of Diseases and Related Health Problems, 10th Revision (ICD-10) (27).

All procedures were in accordance with the Declaration of Helsinki (28). Written informed consent was obtained from all clinicians, while both clinicians and patients provided signed consent for audio recordings of the initial clinical interviews.

### 2.2 Psychometric assessment

The ACSE is a self-completed instrument that was specifically developed to measure clinicians’ subjective experience during the interaction with patients. It consists of 46 items, each rated on a 5-point scale ranging from 0 to 4. The instrument yields scores on five scales, named Tension, Difficulty in Attunement, Engagement, Disconfirmation, and Impotence. The Tension scale consists of items indicating physical tension and clumsiness, reduced spontaneity, and feelings of worry, nervousness, and alarm (e.g., ‘I felt tense in moments of silence’, ‘I maintained a rigid posture’, ‘I was afraid that the patient could act unpredictably’); greater scores indicate higher tension during the visit. The Engagement scale includes items describing the degree of the psychiatrist’s involvement with the patient, such as feelings of boredom, indifference, detachment, lack of attention and, conversely, desire to take care of the patient, and feelings of involvement in the patient-physician relationship, emotional closeness, and tenderness (e.g., ‘I experienced a feeling of tenderness towards the patient’, ‘I felt emotionally close to the patient’). Differently from the items indicating closeness to the patient, the items covering detachment from the patient are reverse-keyed, so that higher scores on this scale indicate greater involvement with the patient. The Disconfirmation scale consists of items describing a failure to establish an authentic relationship with the patient, and feelings of being manipulated, rejected, criticized or devalued by the patient (e.g., ‘I felt depreciated by the patient’, ‘I felt judged by the patient’, ‘I felt rejected by the patient’, ‘I felt that I did not exist for the patient’.); higher scores reflect greater feelings of disconfirmation. The Impotence scale contains items indicating feelings of helplessness, frustration, desolation, emptiness, loneliness, and being drained (e.g., ‘I felt a sense of loneliness’, ‘I felt a sense of emptiness’, ‘At the end of the interview I felt a sense of impotence’); higher scores indicate greater feelings of impotence.

The Difficulty in Attunement scale contains items describing difficulty in establishing emotional contact, being empathic, understanding the patient’s experience, and communicating with the patient. Given the relevance of this scale for the present study, all its items were reported here: ‘At the beginning of the interview I struggled to establish an emotional connection with the patient’; ‘I found it difficult to follow the train of thoughts expressed by the patient’; ‘I perceived a discordance between the way in which the patient experienced some of his/her life events and the way in which I would have experienced them’; ‘I simplified my communication by modifying my usual language’; ‘I carefully chose my words in order not to scare the patient’; ‘I carefully chose my words in order to be easily understood by the patient’; ‘I tempered the tone of my voice in relation to the patient’s state’; ‘There were times when I felt the way in which the patient gave sense to his/her own experiences was alien to me’; ‘I had difficulties in identifying myself with the patient’; ‘I felt a sense of alienation from the patient’. Higher scores reflect greater difficulties in attunement to the patient.

### 2.3 Experimental setting

The experimental setting consisted of three phases: the first clinical interview, administration of the ACSE to clinicians, and listening to the first clinical interview recording by clinicians. Further details on the experimental setting were reported in the **Supplementary Materials**.

Briefly, the clinician, the patient, and a non-interacting specialty psychiatrist attended the first clinical interview, during which clinicians’ EEG and audio recordings were initiated simultaneously. Following the first clinical interview, clinicians completed the Italian version of the ACSE while EEG recording continued. The questionnaire was administered digitally, with items presented sequentially. Finally, after completing the ACSE, the EEG recording was terminated. Clinicians then listened to the audio recording of the first clinical interview and retrospectively identified the temporal occurrence of subjective experiences relevant to each ACSE dimension using a structured form.

**Figure 1** reports a schematic representation of experimental setting, measures derived and analyses conducted.

**Figure 1:**
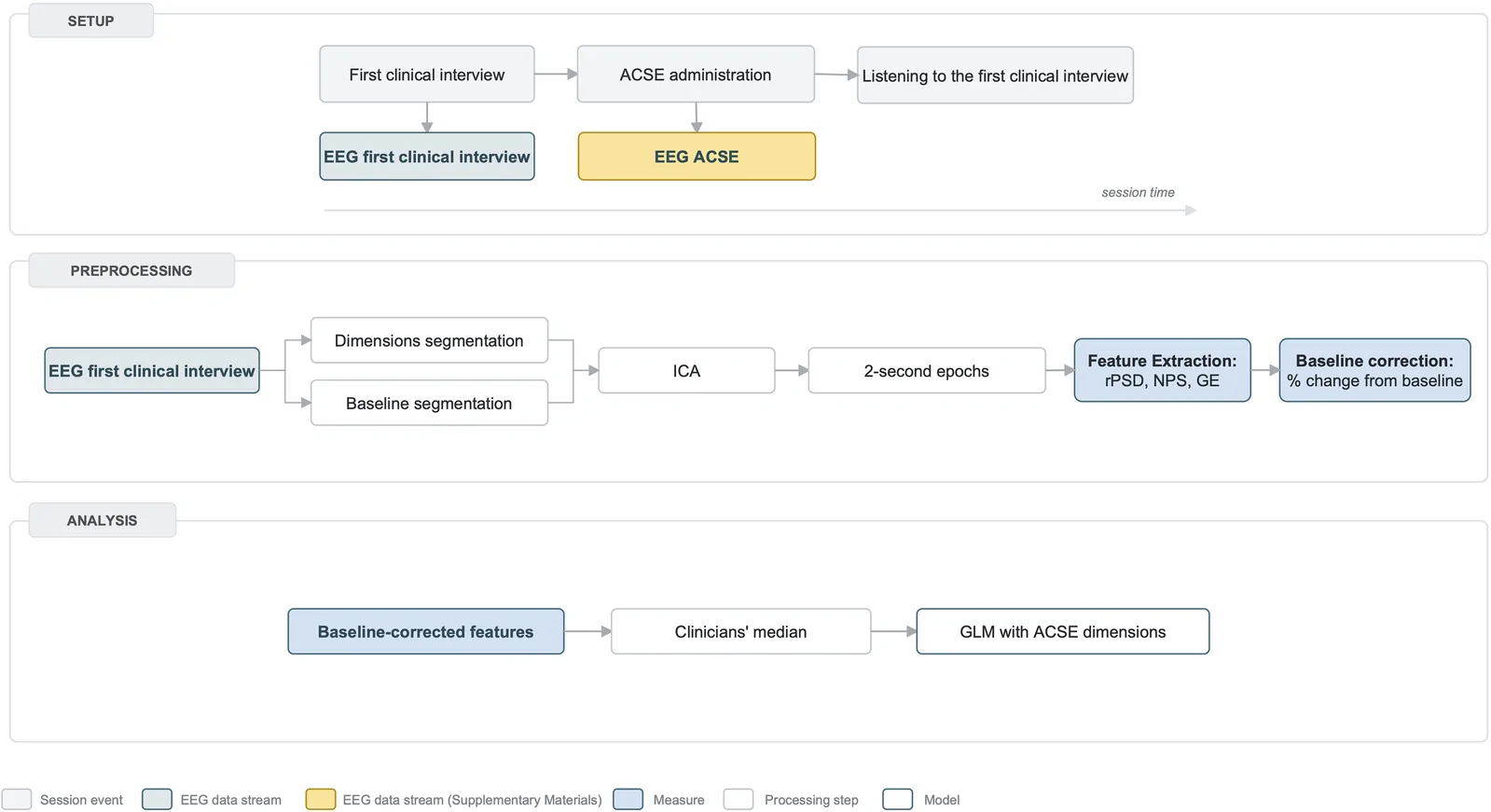
Overview of the full analytic workflow across the three stages of the study: (1) Setup (first clinical interview, ACSE administration, and listening to the audio recording of the first clinical interview); (2) Preprocessing (segmentation of the EEG signal ICA-based artifact removal, epoching, feature extraction - rPSD, NPS, GE -, baseline correction); (3) Analysis, showing the derivation of (clinician-level median values from the baseline-corrected features, input into the GLM with ACSE dimensions). %: percentage; ACSE: Assessment of Clinician's Subjective Experience; EEG: Electroencephalography; ICA: Independent Component Analysis; rPSD: relative Power Spectral Density; NPS: node positive strength; GE: global efficiency; GLM: general linear model.

### 2.4 EEG pipeline

#### 2.4.1 EEG data acquisition

EEG data were collected using a 61-channel Neuro BE PLUS LTM Galileo NT Line 4.50 system (EB Neuro S.p.A.) with sampling frequency set at 1000 Hz, the reference electrode placed frontally, and with electrode impedances maintained below 10 kΩ. Before each session, the EEG signal quality was verified through visual inspection.

#### 2.4.2 EEG data pre-processing

EEG data pre-processing was performed using EEGLAB (v2025.0.0), an open-source MATLAB^®^ 2024b toolbox (The Mathworks, Inc., Natick, Massachusetts, USA) (29). Initial processing steps included band-pass (1–120 Hz) and notch (50 Hz) filters, followed by downsampling to 250 Hz. The following pre-processing pipeline differed depending on the experimental condition. Information on the EEG signal recorded during ACSE administration is provided in the **Supplementary Materials**.

For the first clinical interview dataset, EEG recordings were segmented according to temporal markers provided by the clinician during the listening phase. An artifact-free 120-second segment recorded at the session onset, corresponding to the interview’s information-gathering phase, when the clinician yet reported no ACSE dimension, and allowing control for interview-related neural activity, was extracted and designated as the baseline for this dataset (hereinafter called single-interview baseline). Independent Component Analysis (ICA) was then applied to remove both biological artifacts (primarily ocular and muscular activity) and non-physiological artifacts (such as those resulting from poor electrode contact), selected manually based on a visual inspection of topography and time series (30). Finally, data were epoched into 2-second segments. For each epoch, relative power spectral density (rPSD) and functional connectivity (FC) metrics were computed across standard frequency bands: delta, theta, alpha, beta, low gamma, and high gamma.

#### 2.4.3 EEG feature extraction

PSD was computed by first applying the Fast Fourier Transform (FFT) to each epoch. Power values within specific frequency bands - delta (1–4 Hz), theta (4–8 Hz), alpha (8–12 Hz), beta (12–32 Hz), low gamma (32–70 Hz), and high gamma (70–120 Hz) - were estimated through trapezoidal numerical integration across the corresponding frequency ranges. Normalization was achieved by dividing each band’s power by the total integrated power spanning from 1 Hz to the Nyquist frequency, yielding the rPSD. Finally, for each patient, condition, and frequency band, rPSD values were averaged across all epochs within the entire signal (single-interview baseline dataset) and each ACSE dimension (first clinical interview dataset). The changes induced by the different protocol phases, with respect to each baseline condition, were computed as the baseline-corrected rPSD as reported in Equation 1:

(1) $$rPSD_{condition\_change}\;=\;\frac{\left(rPSD_{condition}\;-\;rPSD_{baseline}\right)}{rPSD_{baseline}}*100$$

The EEG FC was evaluated using the imaginary part of coherency (iCOH), a frequency-domain measure that mitigates volume conduction effects by retaining only the imaginary component of the cross-spectrum (31). All connectivity analyses were conducted using the FieldTrip toolbox (32). Epoched EEG data underwent Fourier transformation via a multitaper approach employing a Hanning window across a frequency range of 1–120 Hz. The absolute value of the imaginary part of the resulting complex-valued spectral estimates was used as iCOH values. Network metrics were extracted using the Brain Connectivity Toolbox (33) in MATLAB. Adjacency matrices were normalized with self-connections set to zero, and two key metrics were computed for each dataset and frequency band: node positive strength (NPS) and global efficiency (GE). NPS, defined as the sum of edge weights connecting to a given node, quantifies the centrality or importance of individual channels within the network. GE measures network-wide information transfer efficiency by calculating the average inverse shortest path length between all node pairs, with higher values indicating more direct and efficient communication. Consistent with the rPSD analysis approach, connectivity metrics were baseline-corrected for all task conditions. Specifically, percentage changes from baseline were computed for each clinician-patient pair across ACSE dimensions and frequency bands during the first clinical interview.

### 2.5 Statistical analysis

#### 2.5.1 Socio-demographic variables and ACSE scores

Descriptive statistics, including means and standard deviations, were computed for participants’ age (clinicians and patients) and for ACSE scores.

#### 2.5.2 EEG

The median values of each EEG metric (rPSD, NPS, and GE) were calculated within each clinician for each ACSE dimension and frequency band.

To assess the relationship between electrophysiological measures and ACSE scores, while controlling for the clinician factor, a General Linear Model (GLM) was fitted separately for each EEG metric, frequency band, and dimension. The model was specified as follows:


EEG metric ∼ 1 + ACSE score + clinician

The clinician variable was inserted into the model as a categorical variable. Data for which the ACSE dimension was not reported or were reported for only one interview per clinician were excluded from the analysis. Given the limited sample size, an uncorrected threshold of p < 0.05 was adopted.

The relationship between EEG metrics and ACSE scores was also investigated during the ACSE administration. Methodology and results are reported in the **Supplementary Materials**.

## 3 Results

### 3.1 Sociodemographic data

In **Table 1** sociodemographic data of clinicians and patients were reported.

**Table 1:** Sociodemographic data of all participants.

Variable	Clinicians (N = 2)	Patients (N = 10)
Sex (M:F)	1:1	4:6
Age (mean ± SD)	39.5 ± 2.5	53.7 ± 18.4
Theoretical background (N)	Cognitive-behavioral (1) No psychotherapy training (1)	–
Psychiatric disorders (N)	–	Mixed anxiety and depressive disorder (7) Unspecified anxiety disorder (3)

F, Females; M, Males; N, Numerosity; SD, Standard Deviation.

Two psychiatrists, a 42-year-old woman (first clinician - C01) and a 37-year-old man (second clinician - C02), were recruited. C01 had no formal psychotherapy training, whereas C02 had formal training in cognitive-behavioral psychotherapy.

Among patients, 4 were males and 6 were females (mean age: 53.7 ± 18.4 years), with a diagnosis of mixed anxiety and depressive disorder (7 patients) and unspecified anxiety disorder (3 patients).

### 3.2 ACSE results

Results of the ACSE questionnaire were reported in **Table 2**.

**Table 2:** Scores of the ACSE questionnaire (reported as mean ± standard deviation).

ACSE dimension	Patients (N = 10)
Tension	1.60 ± 1.62
Difficulty in attunement	8.50 ± 5.12
Engagement	19.20 ± 5.17
Disconfirmation	1.50 ± 2.77
Impotence	2.70 ± 3.13

ACSE, Assessment of Clinician’s Subjective Experience questionnaire; N, Numerosity.

The highest scores were observed for the Difficulty in Attunement (8.50 ± 5.12; range 1-16) and Engagement (19.20 ± 5.17; range 8-27) dimensions, whereas the lowest scores were found for Tension (1.60 ± 1.62; range 0-5) and Disconfirmation (1.50 ± 2.77; range 0-9). The Impotence dimension showed intermediate values (2.70 ± 3.13; range: 0-10) (**Figure 2**).

**Figure 2:**
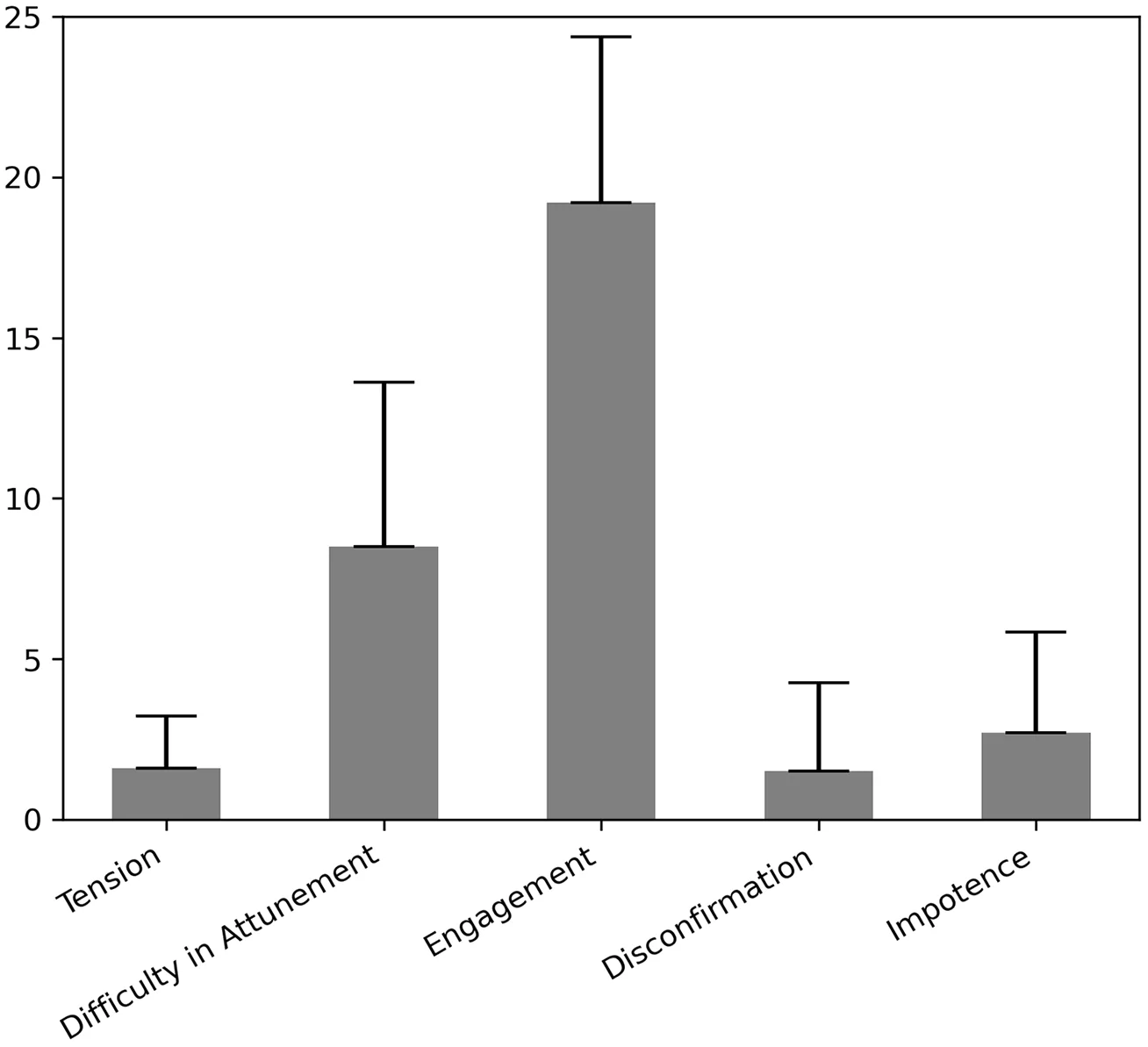
Scores of the ACSE questionnaire (mean and standard deviation). ACSE dimensions are reported on the x-axis, scores of the questionnaire on the y-axis.

Notably, after listening to the first clinical interview recording, clinicians reported that no feelings described by Tension and Impotence dimension emerged during the interactions with the patients. Furthermore, feelings related to the Disconfirmation dimension emerged in only one of the five first clinical interviews for both clinicians, therefore it was excluded from GLM analyses.

### 3.3 Power spectral density results

Analysis of rPSD across ACSE dimensions revealed frequency- and topography-specific patterns (**Figure 3**).

**Figure 3:**
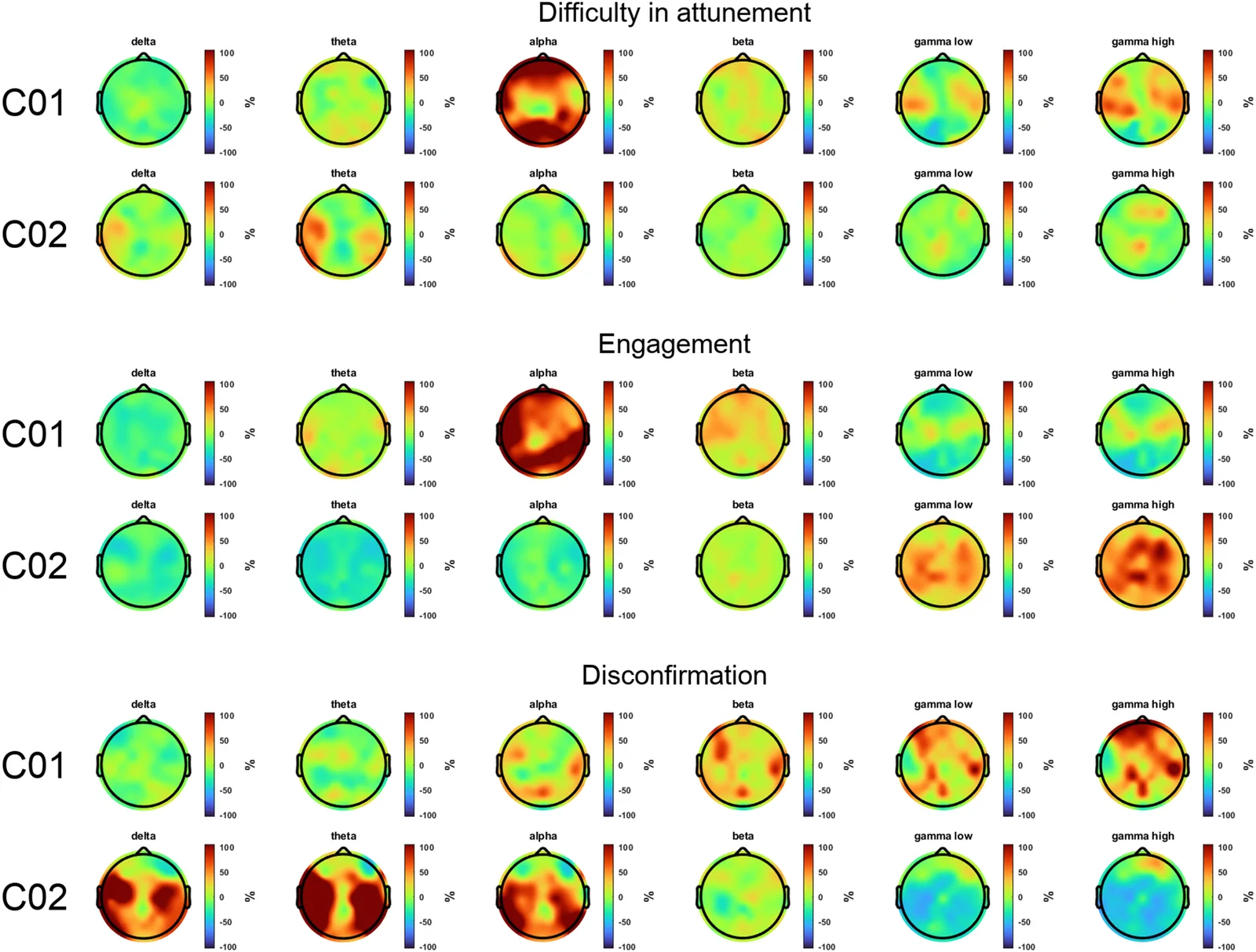
Relative Power Spectral Density (rPSD) analysis of the first clinical interview. The color bar represents the median percentage change in rPSD for each ACSE dimension compared to the baseline. C01, First clinician (female clinician); C02, Second clinician (male clinician).

In the Difficulty in Attunement dimension, C02 exhibited increased power in the delta and theta bands in the temporo-parietal regions, whereas the brain activity of C01 in these lower frequencies remained closer to baseline. Conversely, C01 showed robust and widespread alpha enhancement together with higher beta power, while the brain’s activity of C02 in these bands was comparatively stable. In the gamma range (both low and high), C01 demonstrated increased fronto-temporal gamma power, whereas C02 showed increased fronto-central gamma activity.

A different configuration characterized the Engagement dimension. Here, theta activity increased in the temporal and occipital regions in C01, whereas C02 showed an overall decrease in the same bands. Additionally, C01 displayed marked alpha enhancement and elevated beta power, whereas C02 decreased slightly in the alpha band and remained near baseline in the beta band. In the gamma bands, C02 showed a whole-brain increased activity, whereas the increment was localized in the fronto-temporal and fronto-central regions for C01.

Finally, the Disconfirmation dimension elicited several modulations. C02 showed increased delta and theta power, whereas the brain’s activity of C01 in these bands remained relatively stable. Both clinicians exhibited alpha enhancement, although this effect was more robust and widespread in C02. Beta power increased in C01, while in C02 these frequencies remained closer to baseline. In the gamma bands, C01 demonstrated increased whole-brain activity, whereas C02 showed decreased whole-brain activity.

Overall, C01 exhibited predominant alpha band activity with comparable distribution across other frequency bands for Difficulty in attunement and Engagement, while Disconfirmation showed increased high-frequency band prevalence. On the other hand, C02 demonstrated distinct patterns: low-frequency bands (delta, theta, alpha) predominated for Difficulty in Attunement and Disconfirmation, whereas high-frequency bands (beta, low gamma, high gamma) were more prevalent for Engagement.

#### 3.3.1 Correlation with ACSE

The GLM applied to rPSD data revealed a significant (p <0.05, uncorrected) negative correlation between gamma-band power (both low and high gamma) and the ACSE scores on the Difficulty in Attunement dimension, specifically in bilateral frontal regions, showing that higher Difficulty in Attunement scores were associated with reduced frontal gamma activity (**Figure 4**).

**Figure 4:**
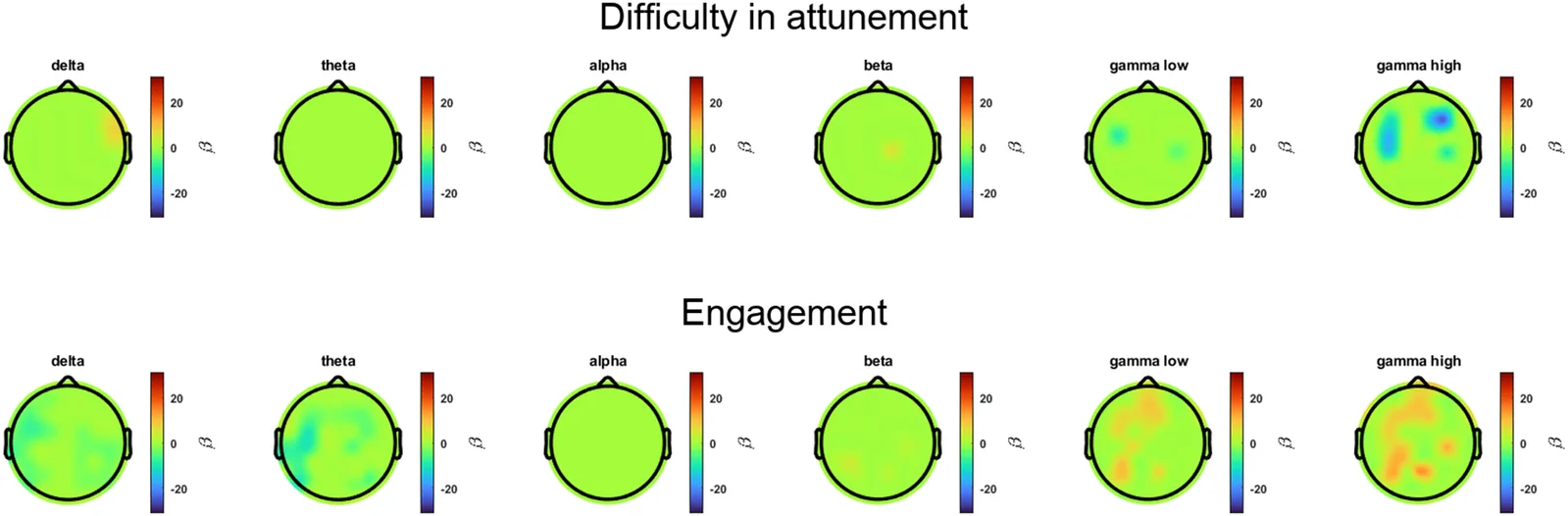
General Linear Model (GLM) significant (p < 0.05, uncorrected) correlation results of relative Power Spectral Density (rPSD) of the first clinical interview. The color bar represents the beta (β) estimates of significant results. The GLM was computed using the *fitglm* function in MATLAB.

The Engagement dimension showed a different pattern. Increasing Engagement scores were associated with enhanced high-frequency power (beta and gamma - low and high - bands) in fronto-parietal regions, alongside reduced low-frequency power (delta and theta bands) in frontal and temporo-parietal areas. None of the effects survived correction for multiple comparisons, using either Bonferroni correction or False Discovery Rate (Benjamini-Hochberg). Further information on the statistics have been reported in the **Supplementary Materials**.

### 3.4 Functional connectivity

Analysis of NPS (**Figure 5**) and GE (**Figure 6**) across ACSE dimensions partially corroborated the rPSD findings.

**Figure 5:**
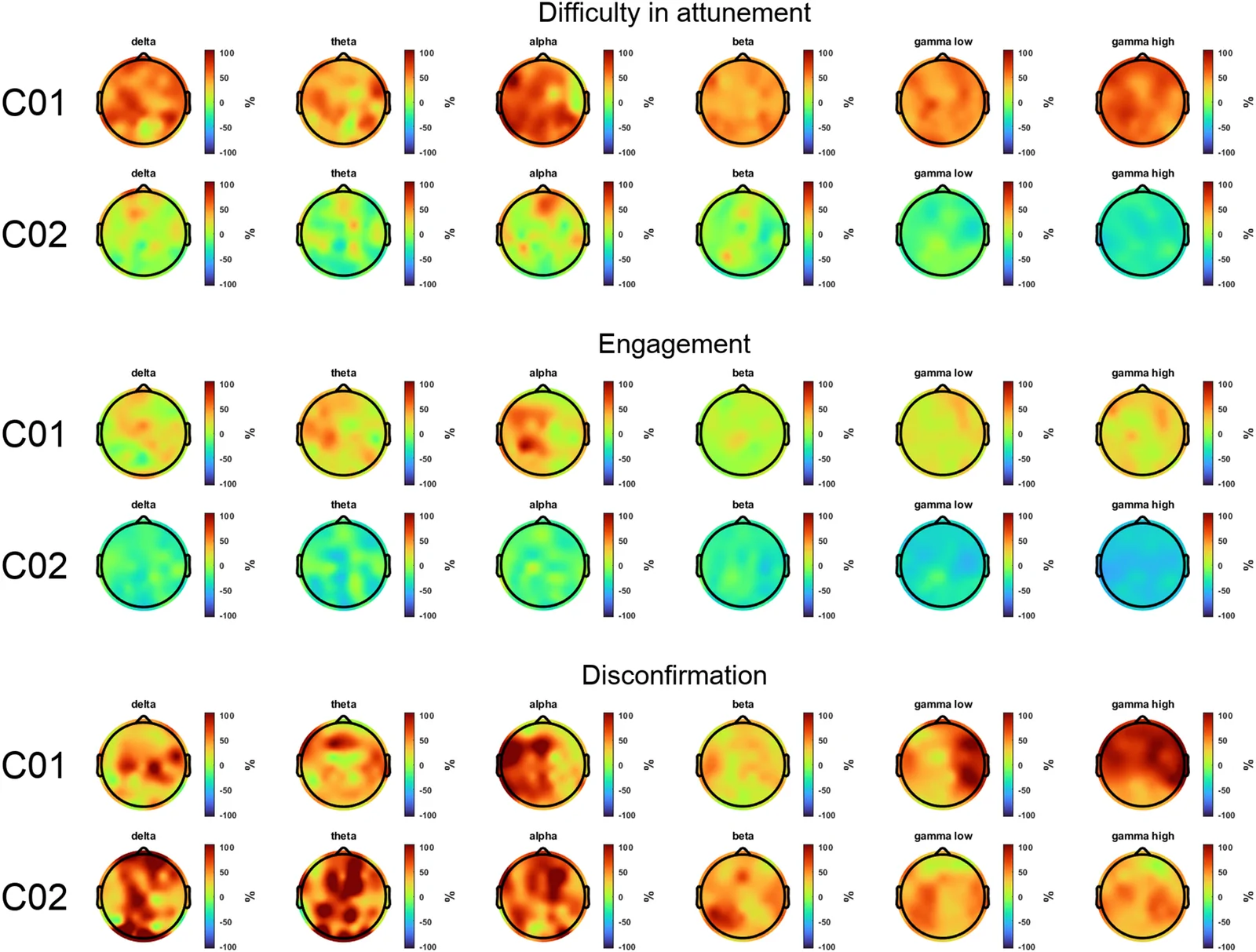
Node Positive Strength (NPS) analysis of the first clinical interview. The color bar represents the median percentage change in the strength of each single node for each ACSE dimension compared to the baseline. C01: First clinician (female clinician); C02: Second clinician (male clinician).

**Figure 6:**
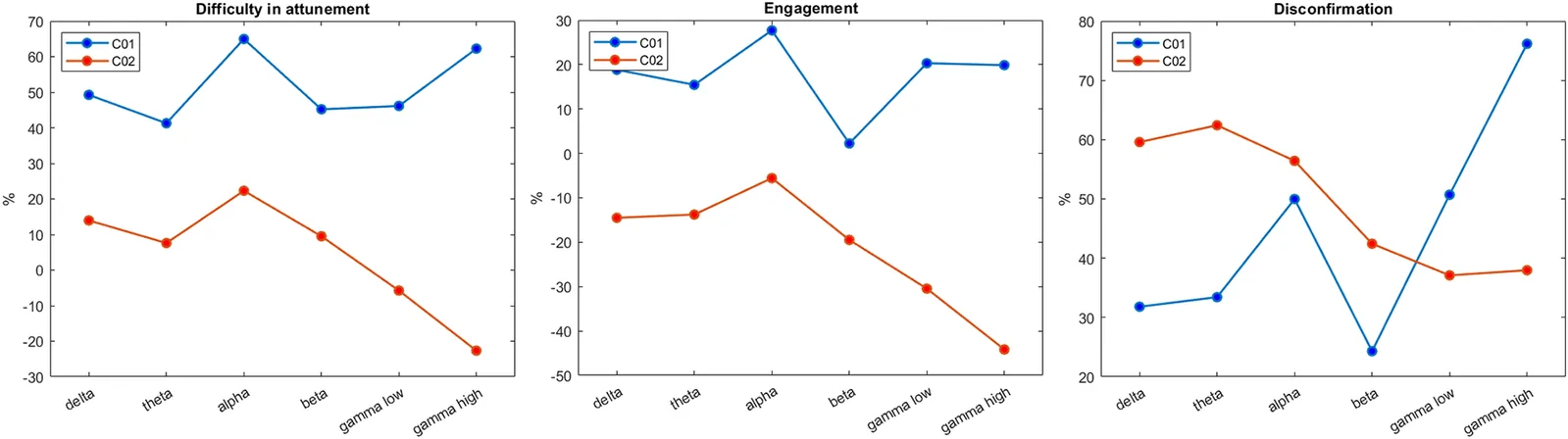
Analysis of global efficiency (GE) of the first clinical interview. On the x-axis, EEG frequency bands are reported. On the y-axis, median percentages of global efficiency with respect to baseline are reported. C01: First clinician (female clinician); C02: Second clinician (male clinician).

In the Difficulty in Attunement and Engagement dimensions, C01 exhibited greater global connectivity, especially in the gamma frequency bands. Notably, in the Engagement dimension, C02 showed connectivity reductions relative to baseline. Similarly, for both Difficulty in Attunement and Engagement dimensions, C01 showed higher GE in all frequency bands.

A frequency-specific pattern emerged for the Disconfirmation dimension, both visible in NPS and GE features. C02 demonstrated higher overall connectivity, predominantly in low-frequency bands (delta, theta, and alpha), whereas C01 showed greater increases in high-frequency connectivity (beta and gamma - low and high).

#### 3.4.1 Correlation with ACSE

For the Difficulty in Attunement dimension, the GLM applied to NPS data revealed a significant (p<0.05, uncorrected) negative association between connectivity positive strength and the corresponding ACSE score. Specifically, higher Difficulty in Attunement scores were associated with reduced connectivity, predominantly in temporo-parietal and occipital regions in the theta band, parietal and central regions in the beta and low-gamma bands, and frontal regions in both low- and high-gamma bands (**Figure 7**). None of the effects survived correction for multiple comparisons, using either Bonferroni correction or False Discovery Rate (Benjamini-Hochberg). No significant associations were observed for the Engagement and Disconfirmation dimensions. Moreover, no significant associations were found for GE. Further information on the statistics have been reported in the **Supplementary Materials**.

**Figure 7:**
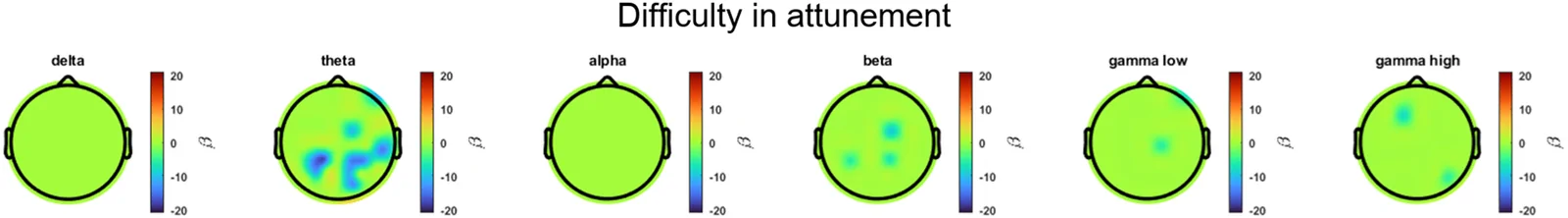
General Linear Model (GLM) significant (p < 0.05, uncorrected) correlation results of Node Positive Strength (NPS) of the first clinical interview. The color bar represents the beta (β) estimates of significant results. The GLM was computed using the *fitglm* function in MATLAB.

## 4 Discussion

To our knowledge, this pilot study is the first to explore the integration of ACSE measures with EEG indices of spectral power and functional connectivity, offering preliminary insights into the potential neurophysiological correlates of clinicians’ subjective experiences during clinical encounters. Questionnaire findings were consistent with previous literature, supporting the instrument’s generalizability in capturing dimension-specific experiential patterns. Importantly, EEG analyses revealed dimension- and frequency-dependent effects across rPSD, NPS and GE. Distinct network configurations between clinicians were observed specifically for two ACSE dimensions (Difficulty in Attunement and Engagement) which markedly emerged during the first clinical interview. Although preliminary, these results suggest that clinicians’ subjective experiences during clinical encounters are not only phenomenological constructs but are also associated with measurable neurophysiological dynamics.

### 4.1 ACSE and intersubjectivity

The ACSE results indicated that the highest scores were observed in the Difficulty in Attunement and Engagement dimensions, whereas the lowest scores were found in the Tension and Disconfirmation dimensions. The Impotence dimension yielded intermediate values, scoring lower than Difficulty in Attunement and Engagement but higher than Tension and Disconfirmation.

Overall, these findings are consistent with previous literature indicating that mood and anxiety disorders are primarily associated with difficulties in emotional attunement, which indeed characterize every clinical encounter with a previously unknown patient (9). Clinicians interacting with patients affected by these conditions also tend to experience lower levels of physical tension and fewer feelings of rejection or failure, as reflected in the minimal or absent experiences related to the Tension and Disconfirmation dimensions during the first clinical interview (9).

Importantly, these results highlight the strong internal consistency and clinical sensitivity of the ACSE in capturing clinicians’ subjective experiences, even within the constraints of a pilot study with a small sample size.

### 4.2 Neurobiological correlates of clinician’s subjective experience

Spectral power and connectivity analyses across the first clinical interview revealed frequency-specific and topographically differentiated patterns. These neurobiological patterns clearly emerged for only two ACSE dimensions: Difficulty in Attunement and Engagement. More restricted evidence was reported for Disconfirmation.

#### 4.2.1 Difficulty in attunement

Difficulty in Attunement appeared to be one of the ACSE dimensions showing the most consistent pattern of EEG modulations across both spectral power and connectivity analyses.

At the spectral level, this dimension was mainly associated with delta- and alpha-band activity, which may be relevant to processes involved in emotional alignment, interoceptive processing, and regulatory control (34, 35). In particular, alpha oscillations have previously been related to states of relaxed alertness and interpersonal attunement (36), whereas delta activity has been discussed in relation to adaptive processes that may support the establishment of emotional contact (37). Notably, the widespread alpha enhancement observed across rPSD and NPS analyses may tentatively suggest the involvement of internal regulatory processes and sustained monitoring of the interpersonal exchange (38, 39). A broadly similar pattern was also observed in GE analyses, in which delta- and alpha-band activity corresponded to two of the most prominent peaks in the connectivity profiles of the two clinicians.

Correlation analyses further suggested a possible involvement of gamma-band activity in Difficulty in Attunement, as higher scores were associated with lower gamma power in frontal regions. Considering the proposed role of gamma oscillations in top-down regulatory and socio-cognitive processes (40, 41), this pattern might be compatible with differences in the recruitment or integration of higher-order processes during moments characterized by greater perceived difficulty in attuning to the patient. Converging evidence was also observed at the connectivity level, where higher Difficulty in Attunement scores were associated with reduced theta-band connectivity in temporo-parietal and occipital regions. Given the involvement of theta oscillations in attentional, visuospatial, and memory-related processes (42), this association may be compatible with differences in the integration of neural processes involved in the processing of relational cues.

Taken together, although these findings remain preliminary, the convergence of spectral and connectivity results may suggest that Difficulty in Attunement represents one of the ACSE dimensions most consistently associated with differentiated neurophysiological patterns.

#### 4.2.2 Engagement

Engagement also appeared to show a relatively consistent pattern across the EEG analyses, with its neurophysiological profile being primarily characterized by modulations in the beta and gamma frequency ranges.

In particular, rPSD analyses revealed increases in beta- and gamma-band activity, together with a positive association between Engagement scores and high-frequency gamma power. Previous studies have implicated beta oscillations in the maintenance of current cognitive or sensorimotor states (43) and in spontaneous cognitive operations (44). In this context, increased beta-band activity may be related to changes in the cognitive or sensorimotor state of the clinician during moments characterized by greater engagement. Beta-band coherence has also been shown to vary according to motor demands (45), raising the possibility that beta activity could, at least in part, be related to the embodied and dynamic nature of clinician-patient interactions. However, given the naturalistic nature of the clinical interaction, beta-band activity may have been influenced by multiple concurrent processes, including cognitive, sensorimotor, and interaction-related factors. Therefore, its specific functional contribution to clinicians’ subjective experience of Engagement cannot be isolated from the present findings.

Similarly, gamma oscillations have been implicated in perceptual encoding, information integration, and the processing of socially salient stimuli (46, 47). The positive association between Engagement scores and gamma-band activity may therefore tentatively suggest a greater involvement of neural processes supporting the processing and integration of interpersonal information during moments characterized by stronger emotional involvement in the first clinical interview.

Connectivity analyses provided additional indications of differentiated network dynamics during Engagement. NPS analyses showed a comparatively less pronounced involvement of beta- and gamma-band connectivity during the interactions with the patient, whereas GE analyses revealed modulations in gamma-band frequencies, with beta activity corresponding to some of the lowest peaks in the connectivity profiles. Although these findings may suggest that spectral power and large-scale connectivity metrics capture partially distinct aspects of the neural dynamics associated with Engagement, their precise functional interpretation remains uncertain.

Overall, although these observations do not allow firm conclusions regarding the underlying cognitive or emotional processes, they may tentatively suggest that Engagement is associated with a differentiated pattern of neural dynamics potentially related to the processing and integration of interpersonal experiences.

#### 4.2.3 Disconfirmation

Compared with Difficulty in Attunement and Engagement, evidence for Disconfirmation was observed in only one of the five first clinical interviews for each clinician. This limited occurrence precluded the inclusion of this dimension in the GLM analyses and, consequently, did not allow for an in-depth investigation of its potential neurobiological correlates. Furthermore, the findings emerging from the spectral power and connectivity analyses were based on interactions involving only one patient for each clinician and, therefore, cannot be considered sufficient to characterize a clear or reproducible pattern of brain activity associated with clinicians’ subjective experience of Disconfirmation.

Specifically, the increased parietal gamma and global beta activity observed in C01 in the rPSD analysis cannot disentangle the potential contribution of these frequency bands to different processes, including motor coordination, intentionality, perceptual integration, and socio-cognitive processing, as suggested by the existing literature (48–50). Similarly, the overlapping activity patterns observed in the two clinicians in the NPS analysis across frequency bands remain difficult to interpret given the limited number of observations. Likewise, the global efficiency profiles cannot be considered sufficiently representative to support specific interpretations regarding neurophysiological patterns potentially associated with the Disconfirmation dimension.

Overall, Disconfirmation could only be explored to a limited extent in the present study, primarily because of constraints inherent to its pilot nature, including the small number of clinicians and patients and the limited occurrence of this dimension across the recorded first clinical interviews. Therefore, the observed findings should be interpreted cautiously and cannot be considered conclusive. Further investigations involving larger and more diverse samples will be necessary to determine whether any of the partially observed patterns represent reproducible neural correlates of this aspect of clinicians’ subjective experience.

### 4.3 Limitations and future directions

Despite its innovative design, this pilot study presents several limitations that warrant consideration.

First, the small sample size, comprising only two clinicians and ten patients, limits the generalizability of the findings and reduces the statistical power to detect subtle effects or perform between-group comparisons. Moreover, the relatively limited clinical severity of the psychiatric diagnoses in the sample may have restricted the expression of certain ACSE dimensions during the first clinical interview. While the dimensions of Difficulty in Attunement and Engagement consistently emerged and could be meaningfully examined, the Tension and Impotence dimensions were not observed in clinician-patient interactions, and Disconfirmation was identified in only one first clinical interview per clinician, precluding a statistically identifiable GLM estimation for this dimension and leading to its exclusion from the analysis. As a result, the limited variability in psychopathological presentations precluded a comprehensive investigation of the neurobiological correlates across all ACSE dimensions. Relatedly, the combination of small sample size and the large number of metric × band × dimension tests limits the robustness of single-model estimates; consistent with this, none of the effects survived correction for multiple comparisons (Bonferroni or False Discovery Rate), and the reported associations should therefore be interpreted as exploratory rather than confirmatory.

Second, although efforts were undertaken to standardize EEG acquisition across ACSE dimensions, variability in interview dynamics and clinician behavior may have introduced uncontrolled sources of bias. In addition, synchronization between ACSE items and EEG segmentation was not automated. To mitigate this limitation, temporal alignment was independently verified by two experimenters to enhance reliability. The single-interview baseline, although designed to control for interview-related neural activity, was not standardized across clinician-patient dyads, and its content, and consequently its stability, may have varied across interviews. Future studies could address this by introducing a fixed set of standardized, neutral questions at the onset of every interview to obtain a more controlled and comparable baseline. Despite these constraints, the overall methodological rigor of the study, particularly the ACSE-guided segmentation procedure and the multimodal EEG analytic approach, provides a solid foundation for future investigations.

Finally, future research should seek to replicate these findings in larger samples encompassing a wider range of clinicians and patient diagnoses, along with the investigation of further EEG measures that could capture the temporal and dynamical richness of clinicians’ subjective experience, such as signal complexity, microstates, and time-resolved connectivity. Such studies would benefit from integrating complementary methodologies, for example, functional near-infrared spectroscopy (fNIRS) to triangulate EEG results and better capture the multidimensional aspects of clinicians’ subjective experience, as well as hyperscanning approaches to examine the bidirectional and dynamic. Additional behavioral and physiological measures (e.g., speech analysis, motion/eye-tracking, heart rate variability) will also be necessary to disentangle the contribution of different interaction-related factors and further characterize the neural correlates associated with clinicians’ subjective experiences. Moreover, although focusing on the first clinical interview allowed us to investigate the emergence of clinicians’ subjective experiences during an initial encounter with the patient, future longitudinal studies should examine whether the identified neural correlates are maintained or modified across repeated clinical interactions and throughout the therapeutic process by combining EEG recordings with the ACSE questionnaire, that can be administered more than once to the same clinician seeing the same patient, as was done to assess the reproducibility of the instrument in the validation studies (6, 12).

### 4.4 Conclusion

This pilot study integrated the ACSE questionnaire with EEG measures for the first time, aiming to investigate potential neurobiological correlates of clinicians’ subjective experiences during the first clinical interview with the patient. The findings provide preliminary evidence that specific experiential dimensions, particularly Difficulty in Attunement and Engagement might be associated with distinct frequency-dependent neural patterns, with variability observed between clinicians.

Although limited by its exploratory design and small sample size, the study suggests that subjective clinical experience might be associated with measurable neural dynamics. These results offer an initial framework for integrating phenomenological and neurobiological approaches to better understand the embodied processes underlying the therapeutic relationship.

## Data Availability

The original contributions presented in the study are included in the article/**Supplementary Material**. Further inquiries can be directed to the corresponding author.
