# Supplementary material for: EEG signature in psychiatrist’s subjective experience: a pilot study
Source: Front Psychiatry. 2026 Oct 5;17:1933800. doi: 10.3389/fpsyt.2026.1933800 (PMC13678462; doi:10.3389/fpsyt.2026.1933800)
Supplement: Supplementary file 1 [file DataSheet1.docx]

**Supplementary materials**

1. **Experimental setting**

*1.1 First clinical interview*

Patients interested in undergoing a first clinical interview were initially contacted by telephone and informed about the study, with emphasis on their secondary role relative to the clinician, who constituted the primary study population. On the day of the interview, patients were asked to arrive approximately 30 minutes prior to the scheduled appointment. Before entering the interview room and meeting the clinician, an experimenter and the specialty psychiatrist responsible for recruitment provided detailed information about the study procedures and research aims. To facilitate a natural clinical interaction, patients were informed about EEG recording and its acquisition procedures and were shown illustrative images of the equipment (e.g., an EEG cap) in advance. Written informed consent and privacy authorization were then obtained.

In parallel, the clinician provided written informed consent and was prepared for EEG recording by an experimenter, who calibrated the equipment and verified signal quality. The EEG equipment was positioned behind the clinician and opposite the room entrance to allow the clinician to maintain a natural posture while minimizing discomfort and to facilitate unobstructed patient entry.

Once preparations were completed, EEG and audio recordings were initiated simultaneously, after which the experimenters left the room and the first clinical interview began. The interview took place in the presence of the clinician, the patient, and the recruiting specialty psychiatrist, who did not interact with the patient and assisted the clinician solely with diagnostic note-taking and treatment documentation. The interview was audio-recorded using a mobile device in airplane mode to minimize interference and lasted approximately 60 minutes.

*1.2 ACSE administration*

After completion of the first clinical interview, the patient left the room and was replaced by two experimenters. The audio recording was then stopped, and the clinician was instructed to complete the Italian version of the ACSE on a computer via Google Forms, while EEG recording continued. Questionnaire items were presented sequentially and in the same order as the original paper version (Pallagrosi et al., 2014). The clinician completed the questionnaire at their own pace and was instructed not to return to previously answered items, a procedure that was essential for accurate EEG time-locking. The ACSE was administered digitally with items presented sequentially to minimize EEG artifacts caused by hand movements and divided attention from responding to multiple items simultaneously.

During ACSE administration, the experimenters coordinated time-marker recording: one marked events within the EEG acquisition system, while the other recorded corresponding timestamps using a mobile phone stopwatch. Time markers were defined by each transition between questionnaire pages, from the appearance of a new item to the final screen, which marked the endpoint of the last interval. Completion of the ACSE required approximately 30 minutes.

*1.3 Listening to the first clinical interview*

After completing the ACSE, the EEG equipment was dismantled, and the clinician listened to the audio recording of the first clinical interview to identify the time points at which each ACSE dimension had manifested. The clinician recorded this information on a paper form listing the definitions of the five ACSE dimensions, including examples of relevant items to facilitate accurate identification. The definitions and item examples were drawn from the original publication by Pallagrosi et al. (2014) and translated into Italian. To preserve confidentiality, the audio was listened to using isolation devices, such as mobile phone headphones, ensuring that sensitive information was not shared with the experimenters. This final phase of the procedure took approximately 20 minutes.

2. **ACSE administration - EEG signal**

**Supplementary Figure S1** reports a schematic representation of the experimental setting, EEG measures, and analyses reported for the ACSE administration session reported in the following sections.

*2.1 EEG data acquisition and pre-processing*

EEG data were collected for each clinician during ACSE administration, as reported in Section 1.2 of SM. Moreover, a 60-second eyes-open resting-state baseline was recorded for each clinician.

EEG data were pre-processed using EEGLAB (v2025.0.0), an open-source MATLAB® 2024b toolbox (The Mathworks, Inc., Natick, Massachusetts, USA) (Delorme & Makeig, 2004). A band-pass (1-120 Hz) and a notch (50 Hz) filter were employed, followed by a downsampling to 250 Hz. Independent Component Analysis (ICA) was performed on the continuous signals. The ACSE EEG recordings were subsequently segmented based on question presentation markers and epoched into 2-second segments. Following the same filtering and ICA steps, the EEG baseline datasets were also epoched into 2-second segments.

*2.2 EEG data analysis*

Relative power spectral density (rPSD) and functional connectivity metrics - node positive strength (NPS) and global efficiency (GE) - were computed, as described in the main manuscript. Specifically, each feature was first calculated for each segment, then averaged across each ACSE question for the EEG recorded during ACSE administration or across all epochs for the baseline datasets. Followingly, the metrics were baseline-corrected as reported in Eq. 1 of the main manuscript.

The median values of each EEG metric (rPSD, NPS, and GE) were calculated within each clinician for each ACSE dimension and frequency band. Moreover, a General Linear Model (GLM) analysis was conducted for each EEG metric, clinician, frequency band, and dimension to evaluate the correlation between neurobiological measures and the ACSE scores, at the net of ACSE items and patient factors. The following model was used:

$EEG metric \sim1 + ACSE item score + ACSE item+ patient$

The patient and ACSE item factors were inserted into the model as categorical variables.

**Supplementary Figure S1** Overview of experimental setting, EEG measures and analyses conducted.

3. **ACSE administration - Results of General Linear Model**

*3.1 rPSD*

Correlation analyses between rPSD and ACSE item scores revealed predominantly weak associations in both clinicians, with most coefficients approaching zero (**Supplementary Figure S2**). Nonetheless, dimension- and frequency-specific patterns emerged.

For Tension, the female clinician (C01 - first clinician) showed positive correlations in parietal and occipital regions within the theta and both low- and high-gamma bands, whereas the male clinician (C02 - second clinician) exhibited positive correlations restricted to frontal regions in the delta band.

Difficulty in Attunement produced in C02 diffuse positive correlations in the theta band and widespread negative correlations in both low- and high-gamma bands. On the other hand, C01 displayed only circumscribed positive associations in occipital regions within the gamma range.

For Engagement, correlations were generally weak or absent in both clinicians, although C02 demonstrated a more evident negative association in the theta band.

Disconfirmation yielded that both clinicians showed positive correlations in frontal, parietal, and occipital regions in the delta, theta, and beta bands, alongside negative correlations in the gamma bands. A positive alpha-band correlation in parietal regions was observed in C02.

Finally, Impotence was characterized by widespread positive correlations in C01 across multiple frequency bands, including delta (occipital regions), theta (parietal regions), and beta (frontal and parietal regions). In C02, positive correlations were confined to frontal regions within the gamma low bands.

**Supplementary Figure S2**. GLM significant (p < 0.05) correlation results of rPSD of ACSE administration.

The color bar represents the beta (β) estimates of significant results. C01: First clinician (female clinician); C02: Second clinician (male clinician).

*3.2 NPS*

Correlation analyses between NPS and ACSE item scores did not reveal consistent significant effects across conditions; however, several clinician- and frequency-specific patterns were identified (**Supplementary Figure S3**).

For Tension, negative correlations emerged in C02, localized to fronto-parietal regions within the theta band.

In Difficulty in Attunement, both clinicians exhibited widespread negative correlations across the scalp and across frequency bands. In C01, these effects were marked by peak reductions in the delta, theta, alpha, and beta ranges.

A distinct pattern characterized Engagement, where C01 showed positive correlations across all frequency bands, predominantly in parietal regions. In the low- and high-gamma bands, these positive associations extended across the scalp.

For Disconfirmation, C01 displayed consistent and widespread negative correlations across all frequency bands.

Finally, Impotence was associated with negative correlations in occipital regions within the theta band in C02.

**Supplementary Figure S3**. GLM significant (p < 0.05) correlation results of NPS of ACSE administration.

The color bar represents the beta (β) estimates of significant results. C01: First clinician (female clinician); C02: Second clinician (male clinician).

*3.3 GE*

Correlation analysis between GE and ACSE item scores revealed negative significant correlation for C01 between all EEG bands and Difficulty in Attunement and Disconfirmation scores (**Supplementary Table S1**). Moreover, differently from C02 (**Supplementary Table S2**), C01 showed a positive correlation between gamma bands and Engagement scores.

**Supplementary Table S1**. GLM significant (p < 0.05) correlation results of GE of ACSE item scores for the first clinician. C01: First clinician (female clinician).

| **C01** | | | | | | |
| --- | --- | --- | --- | --- | --- | --- |
|  | **delta** | **theta** | **alpha** | **beta** | **gamma low** | **gamma high** |
| **Difficulty in Attunement** | 𝛽 = -24.83, p = 0.02 | 𝛽 = -20.90, p = 0.04 | 𝛽 = -21.40, p = 0.02 | 𝛽 = -28.11, p = 0.01 | 𝛽 = -17.14, p = 0.01 | 𝛽 = -13.92, p = 0.01 |
| **Engagement** | ns | ns | ns | ns | 𝛽 = 22.46, p = 0.02 | 𝛽 = 15.75, p = 0.03 |
| **Disconfirmation** | 𝛽 = -86.20, p = 0.03 | 𝛽 = -88.89, p = 0.01 | 𝛽 = -79.57, p = 0.01 | 𝛽 = -96.77, p = 0.01 | 𝛽 = -49.15, p = 0.01 | 𝛽 = -38.28, p = 0.02 |

𝛽: beta score; p: p-value; ns: not significant.

**Supplementary Table S2**. GLM significant (p < 0.05) correlation results of GE of ACSE item scores for the second clinician.

| **C02** | | | | | | |
| --- | --- | --- | --- | --- | --- | --- |
|  | **delta** | **theta** | **alpha** | **beta** | **gamma low** | **gamma high** |
| **Difficulty in attunement** | ns | ns | ns | ns | ns | ns |
| **Engagement** | ns | ns | ns | ns | 𝛽 = -22.18, p = 0.04 | 𝛽 = -21.90, p = 0.04 |
| **Disconfirmation** | ns | ns | ns | ns | ns | ns |

C02: Second clinician (male clinician). 𝛽: beta score; p: p-value; ns: not significant.

4. **First clinical interview - Results of General Linear Model (p-values)**

Epoch counts per clinician and dimension during the first clinical interview are reported in **Supplementary Table S3**.

**Supplementary Table S3**. Mean, standard deviation, and range (minimum-maximum) of the number of epochs identified by the clinician during the first clinical interview for each dimension.

|  | Tension | Difficulty in Attunement | Engagement | Disconfirmation | Impotence |
| --- | --- | --- | --- | --- | --- |
| Number of segments [#] | | | | | |
| C01 | / | 2.0 ± 1.4 (range 1-4) | 2.3 ± 1.5  (range 1-4) | 3.0 ± 0  (range 3-3) | / |
| C02 | / | 1.0 ± 0 (range 1-1) | 1.4 ± 0.9  (range 1-3) | 1.0 ± 0  (range 1-1) | / |

*4.1 rPSD*

Results of correlation analyses between rPSD and ACSE scores during the first clinical interview have been presented in the main text, whereas **Supplementary Tables S4 and S5** show the significant (p<0.05) p-values of Difficulty in Attunement and Engagement. Confidence intervals are presented in **Supplementary Tables S6 and S7.**

**Supplementary Table S4**. GLM significant (p < 0.05) correlation results (p-values) of rPSD and ACSE scores of Difficulty in Attunement.

| **Difficulty in Attunement** | | | | | | |
| --- | --- | --- | --- | --- | --- | --- |
|  | delta | theta | alpha | beta | gamma low | gamma high |
| 'AF7' | ns | ns | ns | ns | ns | ns |
| 'AF3' | ns | ns | ns | ns | ns | ns |
| 'FP1' | ns | ns | ns | ns | ns | ns |
| 'FP2' | ns | ns | ns | ns | ns | ns |
| 'AF4' | ns | ns | ns | ns | ns | ns |
| 'AF8' | ns | ns | ns | ns | ns | ns |
| 'F7' | 0.036 | ns | ns | ns | ns | ns |
| 'F5' | 0.022 | ns | ns | ns | ns | ns |
| 'F3' | ns | ns | ns | ns | ns | 0.045 |
| 'F1' | ns | ns | ns | ns | ns | 0.042 |
| 'F2' | ns | ns | ns | ns | ns | ns |
| 'F4' | ns | ns | ns | ns | ns | 0.039 |
| 'F6' | ns | ns | ns | ns | ns | ns |
| 'F8' | ns | ns | ns | ns | ns | ns |
| 'FT7' | 0.008 | ns | ns | ns | ns | ns |
| 'FC5' | 0.030 | ns | ns | ns | ns | ns |
| 'FC3' | ns | ns | ns | ns | ns | ns |
| 'FC1' | ns | ns | ns | ns | ns | ns |
| 'FC2' | ns | ns | ns | ns | ns | ns |
| 'FC4' | ns | ns | ns | ns | 0.001 | 0.026 |
| 'FC6' | ns | ns | ns | ns | ns | ns |
| 'FT8' | ns | ns | ns | ns | ns | ns |
| 'T3' | ns | ns | ns | ns | ns | ns |
| 'C5' | ns | ns | ns | ns | ns | ns |
| 'C3' | ns | ns | ns | ns | 0.001 | 0.022 |
| 'C1' | ns | ns | ns | 0.049 | ns | ns |
| 'C2' | ns | ns | ns | ns | ns | ns |
| 'C4' | ns | ns | ns | ns | ns | 0.004 |
| 'C6' | ns | ns | ns | ns | ns | ns |
| 'T4' | ns | ns | ns | ns | 0.020 | ns |
| 'TP7' | ns | ns | ns | ns | ns | ns |
| 'CP5' | ns | ns | ns | ns | ns | ns |
| 'CP3' | ns | ns | ns | ns | ns | ns |
| 'CP1' | ns | ns | ns | ns | ns | ns |
| 'CP2' | ns | ns | ns | ns | ns | ns |
| 'CP4' | ns | ns | ns | ns | ns | ns |
| 'CP6' | ns | ns | ns | ns | ns | ns |
| 'TP8' | ns | ns | ns | ns | ns | ns |
| 'T5' | ns | ns | ns | ns | ns | ns |
| 'P5' | ns | ns | ns | ns | ns | ns |
| 'P3' | ns | ns | ns | ns | ns | ns |
| 'P1' | ns | ns | ns | ns | ns | ns |
| 'P2' | ns | ns | ns | ns | ns | ns |
| 'P4' | ns | ns | ns | ns | ns | ns |
| 'P6' | ns | ns | ns | ns | ns | ns |
| 'T6' | ns | ns | ns | ns | ns | ns |
| 'FPZ' | ns | ns | ns | ns | ns | ns |
| 'PO7' | ns | ns | ns | ns | ns | ns |
| 'PO3' | ns | ns | ns | ns | ns | ns |
| 'O1' | ns | ns | ns | ns | ns | ns |
| 'O2' | ns | ns | ns | ns | ns | ns |
| 'PO4' | ns | ns | ns | ns | ns | ns |
| 'PO8' | ns | ns | ns | ns | ns | ns |
| 'OZ' | ns | ns | ns | ns | ns | ns |
| 'AFZ' | ns | ns | ns | ns | ns | ns |
| 'FZ' | ns | ns | ns | ns | ns | ns |
| 'FCZ' | ns | ns | ns | ns | ns | ns |
| 'CZ' | ns | ns | ns | ns | ns | ns |
| 'CPZ' | ns | ns | ns | ns | ns | ns |
| 'PZ' | ns | ns | ns | ns | ns | ns |
| 'POZ' | ns | ns | ns | ns | ns | ns |

ns: not significant.

**Supplementary Table S5**. GLM significant (p < 0.05) correlation results (p-values) of rPSD and ACSE scores of Engagement.

| **Engagement** | | | | | | |
| --- | --- | --- | --- | --- | --- | --- |
|  | delta | theta | alpha | beta | gamma low | gamma high |
| 'AF7' | ns | ns | ns | ns | ns | ns |
| 'AF3' | ns | ns | ns | ns | ns | ns |
| 'FP1' | ns | ns | ns | ns | ns | 0.037 |
| 'FP2' | ns | ns | ns | ns | ns | ns |
| 'AF4' | 0.016 | ns | ns | ns | ns | ns |
| 'AF8' | ns | ns | ns | ns | ns | ns |
| 'F7' | ns | ns | ns | ns | 0.036 | 0.036 |
| 'F5' | ns | ns | ns | ns | ns | ns |
| 'F3' | ns | 0.023 | ns | ns | ns | ns |
| 'F1' | ns | 0.016 | ns | ns | 0.005 | 0.008 |
| 'F2' | ns | 0.049 | ns | ns | 0.026 | 0.015 |
| 'F4' | 0.047 | 0.039 | ns | ns | 0.044 | 0.033 |
| 'F6' | 0.005 | ns | ns | ns | ns | ns |
| 'F8' | ns | ns | ns | ns | ns | 0.018 |
| 'FT7' | ns | ns | ns | ns | ns | ns |
| 'FC5' | ns | ns | ns | ns | ns | ns |
| 'FC3' | ns | 0.049 | ns | ns | ns | ns |
| 'FC1' | 0.047 | ns | ns | ns | ns | ns |
| 'FC2' | 0.036 | ns | ns | ns | ns | ns |
| 'FC4' | 0.029 | 0.026 | ns | ns | 0.035 | 0.026 |
| 'FC6' | 0.018 | ns | ns | ns | ns | ns |
| 'FT8' | ns | ns | ns | ns | ns | ns |
| 'T3' | ns | ns | ns | ns | ns | ns |
| 'C5' | 0.002 | ns | ns | ns | ns | ns |
| 'C3' | 0.025 | ns | ns | 0.028 | ns | 0.044 |
| 'C1' | 0.035 | 0.046 | ns | ns | ns | ns |
| 'C2' | ns | ns | ns | ns | 0.042 | 0.034 |
| 'C4' | ns | 0.036 | ns | ns | ns | 0.045 |
| 'C6' | 0.044 | 0.028 | ns | ns | ns | ns |
| 'T4' | 0.042 | 0.046 | ns | ns | ns | ns |
| 'TP7' | ns | ns | ns | ns | ns | ns |
| 'CP5' | 0.036 | ns | ns | ns | ns | ns |
| 'CP3' | ns | ns | ns | ns | ns | ns |
| 'CP1' | 0.003 | ns | ns | ns | ns | ns |
| 'CP2' | ns | ns | ns | ns | ns | 0.040 |
| 'CP4' | ns | ns | ns | 0.006 | 0.014 | 0.018 |
| 'CP6' | 0.027 | 0.033 | ns | 0.045 | ns | ns |
| 'TP8' | ns | ns | ns | ns | ns | ns |
| 'T5' | 0.031 | ns | ns | ns | ns | ns |
| 'P5' | 0.016 | 0.038 | ns | ns | ns | ns |
| 'P3' | 0.039 | ns | ns | ns | ns | ns |
| 'P1' | 0.028 | ns | ns | 0.007 | 0.035 | 0.042 |
| 'P2' | ns | ns | ns | ns | ns | ns |
| 'P4' | 0.027 | ns | ns | 0.038 | 0.031 | 0.040 |
| 'P6' | 0.034 | 0.045 | ns | ns | 0.024 | 0.024 |
| 'T6' | 0.015 | 0.027 | ns | ns | ns | ns |
| 'FPZ' | ns | ns | ns | ns | 0.043 | 0.028 |
| 'PO7' | ns | ns | ns | ns | ns | ns |
| 'PO3' | 0.046 | 0.045 | ns | ns | ns | ns |
| 'O1' | ns | ns | ns | ns | ns | ns |
| 'O2' | ns | ns | ns | ns | ns | ns |
| 'PO4' | ns | ns | ns | ns | ns | ns |
| 'PO8' | ns | ns | ns | ns | ns | ns |
| 'OZ' | ns | ns | ns | ns | ns | ns |
| 'AFZ' | ns | ns | ns | ns | 0.024 | 0.017 |
| 'FZ' | ns | 0.038 | ns | ns | 0.008 | 0.029 |
| 'FCZ' | ns | ns | ns | ns | 0.021 | ns |
| 'CZ' | ns | ns | ns | ns | ns | ns |
| 'CPZ' | ns | ns | ns | ns | ns | ns |
| 'PZ' | ns | ns | ns | ns | ns | 0.039 |
| 'POZ' | ns | ns | ns | ns | ns | ns |

ns: not significant.

**Supplementary Table S6**. Confidence interval (CI) for General Linear Model (GLM) results of relative Power Spectral Density (rPSD) and Difficulty in Attunement dimension during the first clinical interview. The significant results (p<0.05, uncorrected) are reported in bold.

| **Labels** | **Delta** | | **Theta** | | **Alpha** | | **Beta** | | **Gamma Low** | | **Gamma High** | |
| --- | --- | --- | --- | --- | --- | --- | --- | --- | --- | --- | --- | --- |
| *'AF7'* | -7.97 | 5.88 | -19.13 | 14.22 | -61.09 | 40.45 | -6.04 | 14.08 | -25.82 | 35.51 | -30.27 | 25.42 |
| *'AF3'* | -10.13 | 11.64 | -10.35 | 19.26 | -44.89 | 39.17 | -10.11 | 19.95 | -12.56 | 19.82 | -23.98 | 20.17 |
| *'FP1'* | -5.19 | 9.76 | -10.76 | 13.30 | -56.28 | 47.35 | -14.44 | 21.25 | -21.32 | 21.17 | -34.98 | 21.52 |
| *'FP2'* | -9.14 | 10.11 | -14.05 | 11.31 | -55.00 | 46.02 | -14.56 | 20.15 | -15.88 | 18.15 | -27.09 | 21.15 |
| *'AF4'* | -9.17 | 9.69 | -16.39 | 15.27 | -44.11 | 32.17 | -11.97 | 17.50 | -19.57 | 19.99 | -29.60 | 20.62 |
| *'AF8'* | -14.46 | 11.20 | -14.17 | 8.85 | -58.25 | 43.70 | -16.40 | 24.13 | -32.52 | 34.57 | -41.16 | 32.37 |
| *'F7'* | **0.60** | **9.26** | -1.22 | 21.10 | -28.89 | 22.46 | -9.14 | 7.93 | -23.82 | 2.67 | -43.23 | 4.49 |
| *'F5'* | **1.38** | **8.76** | -4.49 | 13.62 | -20.01 | 14.46 | -9.29 | 10.65 | -24.64 | 5.46 | -45.19 | 5.90 |
| *'F3'* | -7.38 | 15.10 | -5.61 | 20.24 | -29.80 | 16.79 | -10.57 | 5.41 | -28.59 | 5.87 | **-47.99** | **-0.93** |
| *'F1'* | -10.23 | 14.25 | -6.27 | 20.44 | -23.58 | 15.89 | -1.90 | 4.78 | -17.38 | 9.28 | **-29.60** | **-1.00** |
| *'F2'* | -4.67 | 9.08 | -9.29 | 20.64 | -25.44 | 18.74 | -13.33 | 19.17 | -12.30 | 12.26 | -19.35 | 13.08 |
| *'F4'* | -6.76 | 9.76 | -11.40 | 23.53 | -22.50 | 15.36 | -18.97 | 20.38 | -22.77 | 13.50 | **-20.45** | **-1.02** |
| *'F6'* | -6.00 | 14.61 | -8.28 | 19.53 | -44.48 | 19.22 | -12.44 | 12.25 | -15.75 | 10.05 | -23.57 | 7.17 |
| *'F8'* | -6.17 | 10.67 | -17.68 | 29.96 | -42.69 | 22.50 | -10.11 | 10.80 | -13.30 | 3.27 | -23.73 | 0.37 |
| *'FT7'* | **1.21** | **3.74** | -4.91 | 15.18 | -35.08 | 18.79 | -1.26 | 7.49 | -2.86 | 8.60 | -4.36 | 3.41 |
| *'FC5'* | **1.40** | **13.58** | -1.90 | 19.24 | -23.26 | 22.36 | -1.70 | 9.20 | -17.15 | 4.79 | -35.78 | 9.63 |
| *'FC3'* | -8.28 | 17.42 | -12.07 | 22.21 | -30.32 | 19.15 | -6.41 | 4.27 | -20.22 | 2.43 | -29.89 | 1.42 |
| *'FC1'* | -13.97 | 13.76 | -9.83 | 19.13 | -22.08 | 17.37 | -5.99 | 10.41 | -17.82 | 17.55 | -22.87 | 16.54 |
| *'FC2'* | -16.57 | 13.49 | -19.61 | 17.98 | -31.61 | 12.56 | -14.82 | 3.27 | -12.03 | 11.01 | -12.71 | 10.96 |
| *'FC4'* | -16.40 | 29.85 | -20.06 | 42.86 | -22.65 | 19.45 | -19.07 | 15.71 | **-11.04** | **-7.05** | **-29.76** | **-3.75** |
| *'FC6'* | -15.31 | 27.57 | -20.53 | 32.63 | -50.61 | 19.42 | -9.56 | 5.93 | -10.50 | 4.04 | -17.74 | 1.97 |
| *'FT8'* | -14.80 | 15.87 | -23.95 | 33.72 | -58.64 | 39.00 | -1.14 | 9.21 | -18.76 | 30.47 | -20.87 | 24.75 |
| *'T3'* | -14.27 | 13.71 | -14.96 | 19.43 | -27.54 | 14.25 | -2.00 | 19.11 | -20.59 | 61.03 | -21.60 | 65.87 |
| *'C5'* | -4.48 | 11.55 | -14.23 | 32.60 | -23.96 | 12.40 | -10.87 | 8.03 | -12.03 | 3.07 | -20.09 | 3.75 |
| *'C3'* | -9.99 | 14.17 | -23.28 | 29.02 | -35.55 | 28.13 | -6.31 | 7.08 | **-7.64** | **-2.29** | **-15.37** | **-2.50** |
| *'C1'* | -11.81 | 14.92 | -18.56 | 24.61 | -17.03 | 19.71 | **0.05** | **11.64** | -21.65 | 31.88 | -20.71 | 22.01 |
| *'C2'* | -17.63 | 15.80 | -13.69 | 19.08 | -14.57 | 15.84 | -5.10 | 9.55 | -17.18 | 14.62 | -24.19 | 10.95 |
| *'C4'* | -15.66 | 27.56 | -22.42 | 40.90 | -27.79 | 21.03 | -15.85 | 13.84 | -17.76 | 3.99 | **-20.42** | **-9.12** |
| *'C6'* | -11.35 | 22.15 | -23.39 | 32.26 | -43.97 | 18.38 | -10.01 | 9.47 | -10.47 | 15.23 | -16.35 | 9.98 |
| *'T4'* | -19.52 | 31.97 | -25.48 | 40.41 | -45.84 | 25.79 | -6.00 | 6.09 | -4.48 | -0.80 | -17.23 | 5.21 |
| *'TP7'* | -16.00 | 11.81 | -30.21 | 28.56 | -59.30 | 37.09 | -11.66 | 18.51 | **-32.15** | **79.02** | -55.93 | 128.79 |
| *'CP5'* | -8.43 | 13.81 | -23.03 | 23.95 | -43.68 | 27.40 | -8.41 | 10.75 | -6.30 | 20.79 | -10.17 | 26.10 |
| *'CP3'* | -13.12 | 15.18 | -23.34 | 29.60 | -52.18 | 30.69 | -4.34 | 8.73 | -6.70 | 10.11 | -8.86 | 10.64 |
| *'CP1'* | -14.43 | 10.88 | -18.27 | 17.69 | -29.79 | 23.33 | -2.69 | 15.16 | -12.64 | 24.53 | -17.39 | 23.90 |
| *'CP2'* | -7.61 | 15.35 | -15.37 | 18.81 | -28.70 | 22.82 | -8.04 | 9.82 | -27.09 | 31.64 | -36.52 | 26.99 |
| *'CP4'* | -8.93 | 14.38 | -15.01 | 18.32 | -38.86 | 24.40 | -11.95 | 7.54 | -18.02 | 7.45 | -24.61 | 0.55 |
| *'CP6'* | -15.55 | 22.83 | -20.14 | 29.67 | -35.88 | 32.92 | -5.09 | 13.91 | -31.12 | 56.21 | -31.38 | 46.68 |
| *'TP8'* | -19.54 | 23.71 | -27.32 | 39.20 | -44.58 | 34.35 | -4.77 | 12.78 | -23.21 | 51.29 | -22.24 | 45.92 |
| *'T5'* | -5.71 | 12.02 | -17.50 | 35.72 | -37.69 | 24.43 | -11.07 | 10.77 | -21.36 | 29.32 | -29.64 | 42.61 |
| *'P5'* | -13.79 | 11.64 | -18.56 | 28.58 | -23.01 | 19.94 | -1.96 | 8.91 | -11.26 | 33.21 | -16.89 | 45.74 |
| *'P3'* | -9.50 | 12.30 | -13.59 | 24.88 | -26.65 | 17.60 | -1.22 | 9.21 | -6.23 | 11.75 | -8.05 | 9.65 |
| *'P1'* | -6.03 | 11.45 | -6.05 | 13.76 | -42.20 | 18.34 | -1.47 | 4.12 | -16.08 | 17.08 | -20.19 | 12.98 |
| *'P2'* | -0.31 | 5.35 | -7.57 | 12.08 | -43.94 | 10.23 | -4.86 | 4.94 | -15.99 | 17.97 | -17.45 | 17.14 |
| *'P4'* | -3.94 | 10.96 | -13.91 | 22.00 | -47.25 | 25.91 | -7.38 | 6.86 | -16.24 | 18.69 | -27.79 | 29.17 |
| *'P6'* | -11.23 | 16.28 | -20.91 | 31.94 | -46.89 | 32.32 | -6.35 | 7.84 | -18.41 | 30.97 | -29.55 | 44.93 |
| *'T6'* | -14.09 | 18.84 | -28.21 | 37.25 | -49.59 | 34.68 | -4.30 | 6.66 | -22.53 | 46.55 | -35.95 | 69.76 |
| *'FPZ'* | -7.82 | 9.31 | -14.14 | 11.86 | -55.96 | 48.23 | -17.91 | 24.34 | -11.47 | 14.39 | -22.76 | 14.97 |
| *'PO7'* | -10.05 | 15.64 | -11.43 | 17.98 | -46.98 | 24.04 | -30.22 | 12.56 | -24.44 | 13.24 | -57.72 | 18.91 |
| *'PO3'* | -6.71 | 11.24 | -6.33 | 16.21 | -31.88 | 20.73 | -8.10 | 14.19 | -10.06 | 13.32 | -16.09 | 19.02 |
| *'O1'* | -10.67 | 9.92 | -16.94 | 13.97 | -45.06 | 15.59 | -14.72 | 11.02 | -16.12 | 17.35 | -18.96 | 19.86 |
| *'O2'* | -6.98 | 13.30 | -17.26 | 20.02 | -47.79 | 16.80 | -10.09 | 5.36 | -7.28 | 14.74 | -11.81 | 19.45 |
| *'PO4'* | -5.04 | 9.48 | -5.69 | 11.71 | -48.06 | 17.95 | -1.63 | 3.51 | -13.30 | 25.46 | -21.57 | 36.45 |
| *'PO8'* | -10.72 | 14.29 | -14.47 | 27.28 | -35.72 | 22.84 | -2.39 | 8.64 | -9.65 | 30.42 | -16.85 | 41.43 |
| *'OZ'* | -10.50 | 15.21 | -12.41 | 17.11 | -48.28 | 23.28 | -8.95 | 10.48 | -13.49 | 24.86 | -19.18 | 31.97 |
| *'AFZ'* | -7.28 | 9.27 | -6.47 | 14.96 | -31.60 | 28.46 | -9.48 | 17.56 | -16.33 | 17.73 | -29.66 | 22.03 |
| *'FZ'* | -7.13 | 11.58 | -7.36 | 19.30 | -19.62 | 12.85 | -5.88 | 10.09 | -14.17 | 13.80 | -26.29 | 19.59 |
| *'FCZ'* | -7.60 | 6.66 | -7.49 | 14.79 | -20.25 | 12.11 | -5.94 | 7.30 | -12.28 | 12.39 | -15.45 | 15.22 |
| *'CZ'* | -9.31 | 3.17 | -13.89 | 9.93 | -17.41 | 10.71 | -4.61 | 6.27 | -13.71 | 25.04 | -17.93 | 27.36 |
| *'CPZ'* | -8.23 | 7.17 | -13.05 | 11.81 | -27.59 | 16.64 | -7.91 | 12.59 | -21.61 | 29.88 | -30.56 | 29.46 |
| *'PZ'* | -4.76 | 7.74 | -10.33 | 13.92 | -41.48 | 10.67 | -1.00 | 4.42 | -15.94 | 25.96 | -24.38 | 25.55 |
| *'POZ'* | -8.80 | 14.00 | -11.46 | 13.36 | -58.18 | 28.52 | -6.15 | 6.47 | -8.92 | 20.00 | -17.52 | 18.76 |

**Supplementary Table S7**. Confidence interval (CI) for General Linear Model (GLM) results of relative Power Spectral Density (rPSD) and Engagement dimension during the first clinical interview. The significant results (p<0.05, uncorrected) are reported in bold.

| **Labels** | **Delta** | | **Theta** | | **Alpha** | | **Beta** | | **Gamma Low** | | **Gamma High** | |
| --- | --- | --- | --- | --- | --- | --- | --- | --- | --- | --- | --- | --- |
| *'AF7'* | -2.57 | 3.22 | -5.58 | 7.12 | -16.41 | 19.37 | -2.47 | 4.95 | -1.71 | 10.52 | -0.43 | 9.81 |
| *'AF3'* | -4.96 | 0.15 | -7.73 | 0.57 | -10.73 | 7.39 | -0.80 | 3.35 | -1.48 | 7.90 | -4.16 | 11.25 |
| *'FP1'* | -5.18 | 1.15 | -6.44 | 3.24 | -16.13 | 11.83 | -1.30 | 8.70 | -0.05 | 13.24 | **0.64** | **14.69** |
| *'FP2'* | -5.49 | 0.65 | -8.07 | 3.84 | -18.40 | 13.09 | -4.39 | 5.55 | -1.38 | 9.19 | -1.00 | 15.17 |
| *'AF4'* | **-4.85** | **-0.75** | -8.13 | 3.72 | -13.28 | 14.89 | -1.30 | 6.66 | -0.51 | 11.06 | -0.41 | 12.64 |
| *'AF8'* | -4.90 | 1.31 | -7.41 | 7.98 | -20.53 | 25.84 | -5.01 | 8.44 | -4.38 | 11.96 | -2.50 | 17.04 |
| *'F7'* | -1.99 | 2.76 | -10.08 | 3.80 | -13.46 | 13.60 | -3.16 | 3.40 | **0.38** | **8.15** | **0.65** | **13.90** |
| *'F5'* | -3.91 | 0.93 | -8.58 | 2.56 | -10.13 | 7.99 | -2.52 | 2.98 | -1.93 | 16.00 | -2.33 | 21.71 |
| *'F3'* | -5.74 | 0.40 | **-7.71** | **-0.81** | -6.76 | 4.38 | -6.89 | 7.64 | -4.18 | 11.03 | -2.88 | 15.39 |
| *'F1'* | -5.55 | 0.62 | **-6.58** | **-0.99** | -7.86 | 5.68 | -0.83 | 5.88 | **3.25** | **11.38** | **3.28** | **14.10** |
| *'F2'* | -4.36 | 0.25 | **-5.53** | **-0.01** | -6.48 | 5.42 | -3.63 | 4.75 | **1.01** | **11.30** | **2.41** | **15.44** |
| *'F4'* | **-5.33** | **-0.04** | **-8.85** | **-0.33** | -9.61 | 9.06 | -4.87 | 10.20 | **0.23** | **12.77** | **0.85** | **14.11** |
| *'F6'* | **-8.28** | **-2.32** | -11.35 | 0.19 | -16.99 | 21.12 | -1.08 | 4.64 | -0.80 | 16.80 | -0.84 | 20.54 |
| *'F8'* | -4.71 | 0.23 | -14.94 | 1.55 | -25.06 | 26.07 | -6.90 | 4.23 | -1.12 | 7.53 | **1.69** | **12.53** |
| *'FT7'* | -3.33 | 0.94 | -8.81 | 6.74 | -11.03 | 17.96 | -3.41 | 2.13 | -4.66 | 5.50 | -3.77 | 8.39 |
| *'FC5'* | -6.92 | 0.52 | -10.36 | 0.76 | -9.28 | 10.25 | -1.99 | 3.48 | -1.70 | 10.97 | -1.45 | 16.72 |
| *'FC3'* | -7.84 | 0.10 | **-9.63** | **-0.02** | -9.64 | 7.33 | -1.47 | 2.36 | -1.80 | 12.81 | -3.27 | 21.44 |
| *'FC1'* | **-6.28** | **-0.06** | -7.38 | 0.44 | -7.39 | 3.63 | -1.40 | 3.59 | -0.79 | 12.16 | -3.82 | 21.64 |
| *'FC2'* | **-6.58** | **-0.31** | -10.76 | 6.28 | -13.91 | 17.13 | -5.29 | 11.93 | -0.27 | 14.16 | -2.28 | 19.71 |
| *'FC4'* | **-12.45** | **-0.95** | **-18.59** | **-1.65** | -10.71 | 10.58 | -2.98 | 8.05 | **0.67** | **13.23** | **1.36** | **14.66** |
| *'FC6'* | **-11.34** | **-1.56** | -16.30 | 0.51 | -17.39 | 24.34 | -3.55 | 5.77 | -2.97 | 11.12 | -3.48 | 12.80 |
| *'FT8'* | -7.57 | 0.47 | -15.77 | 3.08 | -17.90 | 27.56 | -1.72 | 2.85 | -5.13 | 7.89 | -4.26 | 12.04 |
| *'T3'* | -7.56 | 2.64 | -9.78 | 3.15 | -11.13 | 9.31 | -8.86 | 6.90 | -7.78 | 8.05 | -11.93 | 9.10 |
| *'C5'* | **-6.29** | **-2.42** | -17.83 | 9.99 | -12.58 | 9.60 | -3.85 | 8.10 | -0.77 | 13.68 | -2.57 | 19.07 |
| *'C3'* | **-6.65** | **-0.63** | -12.48 | 0.99 | -10.38 | 10.89 | **0.32** | **3.82** | -0.45 | 14.53 | **0.39** | **21.84** |
| *'C1'* | **-5.98** | **-0.31** | **-10.64** | **-0.12** | -11.13 | 9.11 | -3.51 | 2.11 | -3.37 | 9.91 | -4.15 | 16.96 |
| *'C2'* | -5.40 | 0.75 | -8.35 | 1.52 | -7.53 | 6.87 | -1.36 | 5.92 | **0.36** | **13.14** | **0.87** | **16.05** |
| *'C4'* | -10.43 | 0.29 | **-17.87** | **-0.87** | -10.20 | 9.28 | -2.57 | 6.16 | -1.47 | 11.98 | **0.17** | **11.01** |
| *'C6'* | **-8.40** | **-0.15** | **-14.48** | **-1.19** | -18.19 | 20.69 | -3.94 | 4.68 | -1.47 | 7.85 | -1.78 | 9.16 |
| *'T4'* | **-14.45** | **-0.35** | **-18.69** | **-0.26** | -19.23 | 21.61 | -2.10 | 4.38 | -2.55 | 12.53 | -2.20 | 16.62 |
| *'TP7'* | -6.88 | 0.69 | -14.57 | 0.59 | -13.83 | 10.29 | -4.21 | 3.20 | -5.62 | 11.03 | -5.11 | 13.52 |
| *'CP5'* | **-5.97** | **-0.29** | -9.62 | 0.78 | -11.72 | 13.71 | -2.23 | 5.04 | -2.64 | 12.68 | -4.84 | 17.38 |
| *'CP3'* | -6.15 | 0.12 | -12.12 | 1.40 | -15.60 | 19.18 | -0.12 | 3.84 | -0.67 | 12.90 | -0.88 | 18.78 |
| *'CP1'* | **-4.51** | **-1.50** | -7.69 | 2.03 | -9.11 | 12.04 | -1.78 | 2.63 | -1.11 | 13.07 | -1.63 | 22.47 |
| *'CP2'* | -4.18 | 1.51 | -6.90 | 4.43 | -6.34 | 8.26 | -0.31 | 7.02 | -0.32 | 15.76 | **0.64** | **18.91** |
| *'CP4'* | -5.65 | 0.17 | -8.69 | 0.58 | -7.63 | 10.09 | **1.64** | **6.28** | **2.59** | **15.74** | **2.62** | **18.67** |
| *'CP6'* | **-8.45** | **-0.72** | **-13.55** | **-0.82** | -11.92 | 8.79 | **0.05** | **3.26** | -0.53 | 14.62 | -2.58 | 15.47 |
| *'TP8'* | -9.93 | 0.26 | -18.81 | 0.06 | -18.17 | 12.49 | -2.11 | 3.62 | -2.51 | 11.17 | -3.44 | 14.73 |
| *'T5'* | **-5.85** | **-0.40** | -16.26 | 1.34 | -14.25 | 9.57 | -4.04 | 2.98 | -2.08 | 7.88 | -3.34 | 9.34 |
| *'P5'* | **-5.38** | **-0.80** | **-11.75** | **-0.47** | -9.79 | 6.25 | -1.25 | 2.08 | -0.04 | 11.59 | -0.73 | 14.78 |
| *'P3'* | **-5.27** | **-0.19** | -10.25 | 0.77 | -13.04 | 8.90 | -3.45 | 4.06 | -1.37 | 12.87 | -1.48 | 17.13 |
| *'P1'* | **-4.42** | **-0.36** | -5.37 | 1.98 | -9.31 | 16.97 | **0.89** | **3.57** | **0.73** | **14.17** | **0.54** | **21.95** |
| *'P2'* | -2.60 | 0.63 | -4.95 | 3.99 | -11.50 | 17.89 | -0.06 | 5.05 | -0.53 | 14.54 | -0.32 | 25.09 |
| *'P4'* | **-4.93** | **-0.42** | -8.46 | 0.44 | -11.97 | 13.61 | **0.18** | **4.36** | **1.11** | **16.04** | **0.68** | **21.85** |
| *'P6'* | **-6.97** | **-0.38** | **-13.37** | **-0.20** | -12.20 | 9.68 | -0.08 | 4.36 | **1.45** | **14.22** | **1.61** | **15.67** |
| *'T6'* | **-9.58** | **-1.54** | **-18.20** | **-1.58** | -16.41 | 9.72 | -1.09 | 4.55 | -0.23 | 12.02 | -0.96 | 12.92 |
| *'FPZ'* | -4.00 | 1.22 | -6.31 | 4.74 | -13.41 | 11.52 | -2.71 | 6.98 | **0.22** | **9.87** | **1.02** | **12.19** |
| *'PO7'* | -6.31 | 2.66 | -8.23 | 3.33 | -14.89 | 16.31 | -10.58 | 13.75 | -1.06 | 10.35 | -2.20 | 12.04 |
| *'PO3'* | **-5.23** | **-0.07** | **-5.84** | **-0.08** | -8.62 | 10.03 | -1.09 | 2.95 | -0.65 | 10.83 | -1.97 | 16.01 |
| *'O1'* | -4.63 | 1.19 | -9.93 | 6.42 | -16.64 | 21.48 | -5.53 | 6.81 | -1.37 | 7.63 | -1.53 | 9.93 |
| *'O2'* | -5.88 | 1.21 | -7.55 | 1.78 | -12.53 | 13.62 | -1.89 | 3.59 | -0.22 | 9.75 | -0.25 | 14.50 |
| *'PO4'* | -4.57 | 0.10 | -6.47 | 0.93 | -12.03 | 14.44 | -1.42 | 2.12 | -2.25 | 17.32 | -2.25 | 27.30 |
| *'PO8'* | -5.25 | 0.06 | -7.23 | 1.22 | -10.56 | 9.58 | -0.61 | 3.03 | -1.20 | 12.93 | -0.88 | 16.79 |
| *'OZ'* | -3.74 | 1.71 | -6.56 | 4.01 | -12.09 | 17.81 | -2.62 | 5.18 | -1.14 | 11.71 | -1.38 | 16.01 |
| *'AFZ'* | -4.38 | 0.04 | -6.21 | 0.30 | -8.40 | 5.12 | -1.55 | 4.66 | **1.63** | **16.26** | **2.31** | **16.08** |
| *'FZ'* | -4.84 | 0.23 | **-5.64** | **-0.22** | -6.50 | 5.38 | -1.66 | 4.29 | **2.65** | **11.30** | **1.07** | **13.87** |
| *'FCZ'* | -3.13 | 0.26 | -4.23 | 0.83 | -6.00 | 7.08 | -3.00 | 4.67 | **0.85** | **7.14** | -0.36 | 14.18 |
| *'CZ'* | -2.66 | 0.83 | -4.66 | 3.12 | -5.39 | 7.89 | -3.98 | 5.23 | -1.94 | 8.50 | -2.86 | 20.67 |
| *'CPZ'* | -2.44 | 1.18 | -6.09 | 4.61 | -5.72 | 9.17 | -4.27 | 4.97 | -3.32 | 8.49 | -3.05 | 15.85 |
| *'PZ'* | -3.42 | 1.17 | -6.53 | 4.38 | -11.69 | 17.41 | -1.72 | 2.51 | -1.08 | 13.68 | **1.02** | **26.98** |
| *'POZ'* | -5.88 | 0.86 | -6.66 | 4.16 | -9.69 | 15.96 | -0.80 | 4.33 | -1.79 | 15.15 | -0.63 | 22.37 |

*4.2 NPS*

Results of correlation analyses between rPSD and ACSE scores during the first clinical interview have been presented in the main text, whereas **Supplementary Table S8** shows the significant (p<0.05) p-values of Difficulty in Attunement and Engagement. Confidence intervals are presented in **Supplementary Tables S9 and S10.**

**Supplementary Table S8**. GLM significant (p < 0.05) correlation results (p-values) of rPSD and ACSE scores of Difficulty in Attunement.

| **Difficulty in Attunement** | | | | | | |
| --- | --- | --- | --- | --- | --- | --- |
|  | delta | theta | alpha | beta | gamma low | gamma high |
| 'AF7' | ns | 0.040 | ns | ns | 0.037 | ns |
| 'AF3' | ns | ns | ns | ns | ns | ns |
| 'FP1' | ns | ns | ns | ns | ns | ns |
| 'FP2' | ns | ns | ns | ns | ns | ns |
| 'AF4' | ns | ns | ns | ns | ns | ns |
| 'AF8' | ns | ns | ns | ns | ns | ns |
| 'F7' | ns | ns | ns | ns | ns | ns |
| 'F5' | ns | ns | ns | ns | ns | ns |
| 'F3' | ns | ns | ns | ns | ns | ns |
| 'F1' | ns | ns | ns | ns | ns | ns |
| 'F2' | ns | ns | ns | ns | ns | 0.026 |
| 'F4' | ns | ns | ns | ns | ns | ns |
| 'F6' | ns | ns | ns | ns | ns | ns |
| 'F8' | ns | ns | ns | ns | ns | ns |
| 'FT7' | ns | ns | ns | ns | ns | ns |
| 'FC5' | ns | ns | ns | ns | ns | ns |
| 'FC3' | ns | ns | ns | ns | ns | ns |
| 'FC1' | ns | 0.013 | ns | 0.048 | ns | ns |
| 'FC2' | ns | ns | ns | ns | ns | ns |
| 'FC4' | ns | ns | ns | ns | ns | ns |
| 'FC6' | ns | ns | ns | ns | ns | ns |
| 'FT8' | ns | ns | ns | ns | ns | ns |
| 'T3' | ns | ns | ns | ns | ns | ns |
| 'C5' | ns | 0.021 | ns | ns | ns | ns |
| 'C3' | ns | ns | ns | ns | ns | ns |
| 'C1' | ns | ns | ns | ns | 0.025 | ns |
| 'C2' | ns | ns | ns | ns | ns | ns |
| 'C4' | ns | ns | ns | ns | ns | ns |
| 'C6' | ns | ns | ns | ns | ns | ns |
| 'T4' | ns | ns | ns | ns | ns | ns |
| 'TP7' | ns | ns | ns | ns | ns | ns |
| 'CP5' | ns | ns | ns | ns | ns | ns |
| 'CP3' | ns | 0.042 | ns | ns | ns | ns |
| 'CP1' | ns | 0.025 | ns | 0.022 | ns | ns |
| 'CP2' | ns | 0.030 | ns | ns | ns | ns |
| 'CP4' | ns | 0.021 | ns | 0.042 | ns | ns |
| 'CP6' | ns | ns | ns | ns | ns | ns |
| 'TP8' | ns | ns | ns | ns | ns | ns |
| 'T5' | ns | ns | ns | ns | ns | ns |
| 'P5' | ns | ns | ns | ns | ns | 0.049 |
| 'P3' | ns | ns | ns | ns | ns | ns |
| 'P1' | ns | 0.048 | ns | ns | ns | ns |
| 'P2' | ns | ns | ns | ns | ns | ns |
| 'P4' | ns | 0.033 | ns | ns | ns | ns |
| 'P6' | ns | ns | ns | ns | ns | ns |
| 'T6' | ns | ns | ns | ns | ns | ns |
| 'FPZ' | ns | ns | ns | ns | ns | ns |
| 'PO7' | ns | ns | ns | ns | ns | ns |
| 'PO3' | ns | 0.022 | ns | ns | ns | ns |
| 'O1' | ns | ns | ns | ns | ns | ns |
| 'O2' | ns | ns | ns | ns | ns | ns |
| 'PO4' | ns | ns | ns | ns | ns | ns |
| 'PO8' | ns | ns | ns | ns | ns | ns |
| 'OZ' | ns | ns | ns | ns | ns | ns |
| 'AFZ' | ns | ns | ns | ns | ns | ns |
| 'FZ' | ns | ns | ns | ns | ns | ns |
| 'FCZ' | ns | ns | ns | ns | ns | ns |
| 'CZ' | ns | ns | ns | ns | ns | ns |
| 'CPZ' | ns | ns | ns | ns | ns | ns |
| 'PZ' | ns | ns | ns | ns | ns | ns |
| 'POZ' | ns | ns | ns | ns | ns | ns |

ns: not significant.

**Supplementary Table S9**. Confidence interval (CI) for General Linear Model (GLM) results of Node Positive Strength (NPS) and Difficulty in Attunement dimension during the first clinical interview. The significant results (p<0.05, uncorrected) are reported in bold.

| **Labels** | **Delta** | | **Theta** | | **Alpha** | | **Beta** | | **Gamma Low** | | **Gamma High** | |
| --- | --- | --- | --- | --- | --- | --- | --- | --- | --- | --- | --- | --- |
| *'AF7'* | -39.58 | 20.16 | **-21.58** | **-0.99** | -20.40 | 15.55 | -20.91 | 9.39 | **-14.10** | **-0.82** | -17.52 | 8.60 |
| *'AF3'* | -24.68 | 13.96 | -16.79 | 6.19 | -31.49 | 21.84 | -25.46 | 13.95 | -16.42 | 7.49 | -14.11 | 2.82 |
| *'FP1'* | -32.48 | 18.37 | -19.59 | 9.94 | -25.58 | 20.45 | -22.94 | 10.38 | -17.21 | 6.58 | -19.39 | 3.49 |
| *'FP2'* | -35.89 | 4.87 | -19.66 | 4.37 | -25.84 | 13.08 | -22.50 | 10.72 | -19.18 | 8.48 | -21.56 | 5.43 |
| *'AF4'* | -26.55 | 10.66 | -20.16 | 15.20 | -25.20 | 17.06 | -19.31 | 9.59 | -14.19 | 4.08 | -15.56 | 4.96 |
| *'AF8'* | -24.42 | 13.84 | -26.78 | 7.23 | -27.79 | 10.44 | -22.06 | 13.88 | -11.28 | 7.59 | -10.99 | 5.84 |
| *'F7'* | -25.67 | 24.99 | -27.33 | 15.16 | -21.75 | 14.34 | -21.99 | 11.34 | -21.19 | 8.94 | -24.35 | 13.84 |
| *'F5'* | -27.39 | 27.10 | -14.98 | 10.53 | -22.49 | 10.77 | -26.29 | 15.76 | -20.58 | 9.90 | -15.41 | 6.59 |
| *'F3'* | -21.43 | 16.86 | -15.99 | 14.27 | -26.25 | 22.31 | -20.14 | 7.94 | -12.91 | 10.07 | -9.58 | 3.44 |
| *'F1'* | -28.46 | 20.05 | -17.37 | 7.74 | -27.59 | 12.37 | -24.83 | 11.11 | -15.82 | 4.48 | -17.38 | 2.98 |
| *'F2'* | -34.98 | 15.92 | -33.22 | 16.06 | -37.90 | 18.95 | -26.87 | 12.54 | -14.23 | 2.89 | **-13.99** | **-1.83** |
| *'F4'* | -30.35 | 13.59 | -26.31 | 15.58 | -34.74 | 19.71 | -17.92 | 7.42 | -10.66 | 0.92 | -16.64 | 8.03 |
| *'F6'* | -25.66 | 25.56 | -17.87 | 6.60 | -36.14 | 23.96 | -22.40 | 13.14 | -5.79 | 10.54 | -8.37 | 8.79 |
| *'F8'* | -12.98 | 4.66 | -23.31 | 18.94 | -41.91 | 25.95 | -14.94 | 6.08 | -16.36 | 12.59 | -21.06 | 12.52 |
| *'FT7'* | -15.14 | 12.38 | -25.45 | 5.67 | -29.40 | 35.80 | -14.11 | 12.97 | -11.07 | 10.35 | -11.79 | 10.82 |
| *'FC5'* | -21.94 | 4.08 | -27.10 | 8.25 | -13.44 | 0.38 | -17.17 | 6.09 | -17.58 | 5.66 | -19.10 | 12.17 |
| *'FC3'* | -24.36 | 14.50 | -15.00 | 1.33 | -28.02 | 38.32 | -17.57 | 10.54 | -9.83 | 8.66 | -11.49 | 8.05 |
| *'FC1'* | -35.15 | 19.10 | **-16.90** | **-4.15** | -16.94 | 4.67 | **-18.98** | **-0.18** | -14.09 | 1.27 | -20.17 | 4.75 |
| *'FC2'* | -30.93 | 13.36 | -26.67 | 13.24 | -34.09 | 23.93 | -14.85 | 6.83 | -12.39 | 3.55 | -16.86 | 5.35 |
| *'FC4'* | -35.20 | 12.57 | -32.40 | 18.18 | -36.59 | 18.81 | -13.37 | 6.60 | -15.40 | 8.48 | -20.55 | 13.50 |
| *'FC6'* | -33.45 | 16.55 | -31.12 | 18.86 | -42.62 | 16.92 | -24.08 | 11.24 | -17.65 | 8.68 | -17.93 | 8.68 |
| *'FT8'* | -36.98 | 27.16 | -28.95 | 19.75 | -36.02 | 5.19 | -21.04 | 7.65 | -22.82 | 12.59 | -20.07 | 10.18 |
| *'T3'* | -36.71 | 24.86 | -29.35 | 2.03 | -27.07 | 9.72 | -20.73 | 9.80 | -14.06 | 10.02 | -15.49 | 10.51 |
| *'C5'* | -21.72 | 9.12 | **-25.44** | **-4.27** | -23.62 | 10.66 | -20.49 | 7.66 | -11.22 | 8.73 | -10.13 | 8.54 |
| *'C3'* | -17.83 | 8.94 | -25.39 | 0.93 | -25.23 | 19.77 | -13.97 | 3.88 | -14.79 | 7.14 | -11.93 | 9.45 |
| *'C1'* | -54.94 | 35.65 | -21.55 | 3.34 | -21.88 | 18.85 | -20.43 | 5.02 | **-10.18** | **-1.37** | -14.82 | 5.02 |
| *'C2'* | -28.43 | 15.29 | -25.93 | 6.21 | -34.50 | 28.61 | -13.85 | 9.59 | -9.68 | 10.71 | -8.44 | 5.26 |
| *'C4'* | -32.79 | 10.74 | -32.27 | 9.51 | -36.72 | 23.21 | -14.41 | 5.94 | -18.10 | 11.95 | -22.32 | 16.75 |
| *'C6'* | -31.87 | 21.77 | -14.87 | 5.14 | -25.90 | 0.98 | -21.71 | 9.55 | -20.85 | 10.56 | -20.07 | 11.88 |
| *'T4'* | -27.24 | 19.95 | -25.11 | 11.54 | -27.71 | 13.22 | -20.17 | 7.98 | -15.29 | 4.25 | -11.93 | 5.12 |
| *'TP7'* | -24.30 | 7.68 | -23.48 | 2.84 | -13.51 | 5.00 | -21.20 | 10.35 | -20.04 | 9.00 | -21.00 | 3.85 |
| *'CP5'* | -35.71 | 6.02 | -28.61 | 3.31 | -23.90 | 21.02 | -18.19 | 5.56 | -23.86 | 9.26 | -16.94 | 5.05 |
| *'CP3'* | -28.62 | 7.34 | **-27.82** | **-0.95** | -35.78 | 23.94 | -20.96 | 7.69 | -18.00 | 6.93 | -16.87 | 6.68 |
| *'CP1'* | -27.55 | 8.17 | **-23.75** | **-3.17** | -26.45 | 14.78 | **-11.76** | **-1.89** | -14.52 | 4.46 | -11.54 | 5.43 |
| *'CP2'* | -27.77 | 5.81 | **-31.45** | **-3.18** | -42.74 | 26.25 | -16.37 | 4.17 | -13.17 | 8.39 | -17.07 | 9.37 |
| *'CP4'* | -19.61 | 4.54 | **-21.38** | **-3.57** | -57.19 | 19.74 | **-11.11** | **-0.41** | -16.79 | 11.71 | -22.69 | 14.60 |
| *'CP6'* | -30.39 | 9.97 | -23.47 | 8.47 | -31.12 | 22.38 | -20.46 | 9.45 | -20.89 | 10.93 | -20.10 | 9.24 |
| *'TP8'* | -26.33 | 10.01 | -30.72 | 0.20 | -30.64 | 10.55 | -21.49 | 13.26 | -19.13 | 6.37 | -20.52 | 10.18 |
| *'T5'* | -23.91 | 12.43 | -26.69 | 6.34 | -40.09 | 14.27 | -22.30 | 11.18 | -16.25 | 10.60 | -11.93 | 5.17 |
| *'P5'* | -35.19 | 3.88 | -25.45 | 3.93 | -21.07 | 20.46 | -21.61 | 12.71 | -16.10 | 8.84 | **-11.31** | **-0.06** |
| *'P3'* | -23.92 | 17.43 | -27.12 | 5.06 | -32.11 | 24.76 | -21.10 | 7.63 | -16.20 | 6.50 | -11.17 | 0.62 |
| *'P1'* | -21.71 | 5.87 | **-23.33** | **-0.19** | -25.18 | 13.05 | -17.57 | 6.31 | -14.81 | 5.72 | -11.77 | 4.54 |
| *'P2'* | -25.49 | 8.41 | -29.04 | 1.64 | -28.36 | 8.89 | -20.86 | 8.98 | -20.33 | 10.28 | -15.95 | 4.73 |
| *'P4'* | -28.89 | 6.62 | **-21.51** | **-1.78** | -26.07 | 2.27 | -26.87 | 8.30 | -17.22 | 5.84 | -17.63 | 5.93 |
| *'P6'* | -35.84 | 21.29 | -23.21 | 7.38 | -23.51 | 18.72 | -19.62 | 7.30 | -16.67 | 5.70 | -16.42 | 3.84 |
| *'T6'* | -34.37 | 17.93 | -25.79 | 5.41 | -23.67 | 15.73 | -19.97 | 11.22 | -15.63 | 4.73 | -13.71 | 3.68 |
| *'FPZ'* | -30.29 | 9.67 | -20.39 | 4.07 | -22.55 | 19.10 | -21.38 | 10.71 | -19.67 | 9.69 | -20.34 | 4.82 |
| *'PO7'* | -25.03 | 17.51 | -17.52 | 5.18 | -30.95 | 19.80 | -22.57 | 15.63 | -21.54 | 10.15 | -19.64 | 11.56 |
| *'PO3'* | -15.32 | 7.51 | **-23.59** | **-3.67** | -21.04 | 11.99 | -20.74 | 8.98 | -17.79 | 8.20 | -11.26 | 3.43 |
| *'O1'* | -35.34 | 25.01 | -24.95 | 5.76 | -19.46 | 10.40 | -21.39 | 13.12 | -17.97 | 14.36 | -16.68 | 8.50 |
| *'O2'* | -26.61 | 7.67 | -19.26 | 5.63 | -24.95 | 3.18 | -19.77 | 10.04 | -14.80 | 7.72 | -20.61 | 6.94 |
| *'PO4'* | -27.01 | 9.27 | -25.47 | 1.69 | -24.54 | 3.29 | -21.71 | 9.62 | -18.11 | 7.70 | -19.93 | 4.66 |
| *'PO8'* | -30.31 | 10.93 | -26.67 | 9.08 | -17.76 | 3.91 | -17.82 | 9.25 | -15.88 | 7.25 | -18.85 | 5.57 |
| *'OZ'* | -21.97 | 17.55 | -21.88 | 16.78 | -24.35 | 13.89 | -17.56 | 11.72 | -14.15 | 9.16 | -19.31 | 7.59 |
| *'AFZ'* | -27.56 | 18.65 | -22.01 | 8.13 | -29.26 | 14.63 | -23.27 | 12.12 | -16.70 | 12.01 | -18.00 | 5.71 |
| *'FZ'* | -30.18 | 14.54 | -32.58 | 15.07 | -40.07 | 17.46 | -21.31 | 11.76 | -15.91 | 10.61 | -16.50 | 1.18 |
| *'FCZ'* | -27.20 | 13.02 | -27.33 | 8.89 | -30.26 | 8.76 | -16.87 | 8.92 | -11.63 | 2.55 | -19.09 | 1.51 |
| *'CZ'* | -29.50 | 5.49 | -16.97 | 0.71 | -23.04 | 16.78 | -16.74 | 7.19 | -13.38 | 6.51 | -17.86 | 2.25 |
| *'CPZ'* | -26.76 | 5.10 | -22.05 | 5.50 | -29.74 | 15.76 | -14.34 | 1.00 | -13.12 | 5.70 | -13.42 | 5.66 |
| *'PZ'* | -16.42 | 1.80 | -18.28 | 3.07 | -30.29 | 17.54 | -18.20 | 8.19 | -15.37 | 6.12 | -16.82 | 5.36 |
| *'POZ'* | -18.92 | 7.44 | -21.83 | 10.27 | -28.55 | 19.84 | -16.87 | 9.40 | -15.05 | 6.40 | -15.20 | 4.87 |

**Supplementary Table S10**. Confidence interval (CI) for General Linear Model (GLM) results of Node Positive Strength (NPS) and Engagement dimension during the first clinical interview. The significant results (p<0.05, uncorrected) are reported in bold.

| **Labels** | **Delta** | | **Theta** | | **Alpha** | | **Beta** | | **Gamma Low** | | **Gamma High** | |
| --- | --- | --- | --- | --- | --- | --- | --- | --- | --- | --- | --- | --- |
| *'AF7'* | -8.18 | 3.30 | -6.03 | 3.82 | -9.20 | 2.57 | -7.35 | 4.33 | -5.16 | 0.86 | -5.52 | 2.71 |
| *'AF3'* | -8.54 | 3.64 | -6.15 | 2.34 | -12.93 | 7.79 | -9.71 | 5.16 | -5.42 | 0.50 | -5.17 | 3.58 |
| *'FP1'* | -10.20 | 3.04 | -7.78 | 0.33 | -13.28 | 4.59 | -9.95 | 3.53 | -6.28 | -0.04 | -6.77 | 3.47 |
| *'FP2'* | -12.45 | 8.32 | -7.62 | 2.69 | -10.71 | 4.58 | -7.91 | 3.07 | -5.70 | 0.88 | -5.93 | 3.53 |
| *'AF4'* | -8.88 | 3.73 | -6.79 | 0.78 | -10.52 | 2.96 | -7.69 | 2.51 | -5.65 | 1.08 | -5.16 | 3.56 |
| *'AF8'* | -8.93 | 2.01 | -5.55 | 2.97 | -8.31 | 3.49 | -8.53 | 2.71 | -7.44 | -0.58 | -5.67 | 2.00 |
| *'F7'* | -8.41 | 2.64 | -9.77 | 5.38 | -10.25 | 4.26 | -6.63 | 2.28 | -6.09 | 2.56 | -6.32 | 2.77 |
| *'F5'* | -6.87 | 3.36 | -6.41 | 4.18 | -8.60 | 5.07 | -8.20 | 6.54 | -5.48 | 4.44 | -4.75 | 4.13 |
| *'F3'* | -6.83 | 2.70 | -5.52 | 1.88 | -7.60 | 1.58 | -6.67 | 3.60 | -5.73 | 2.57 | -5.09 | 3.51 |
| *'F1'* | -8.00 | 4.91 | -5.66 | 2.65 | -8.68 | 3.48 | -8.03 | 3.96 | -4.57 | 1.12 | -5.32 | 4.18 |
| *'F2'* | -10.34 | 5.65 | -5.72 | 3.99 | -7.15 | 5.39 | -7.89 | 4.85 | -4.30 | 2.04 | -4.43 | 4.43 |
| *'F4'* | -7.06 | 3.88 | -4.61 | 2.94 | -5.23 | 5.27 | -6.69 | 3.83 | -4.59 | 1.29 | -4.57 | 3.07 |
| *'F6'* | -7.66 | 1.57 | -3.42 | 4.55 | -4.71 | 7.08 | -6.50 | 2.95 | -7.87 | 2.26 | -5.39 | 3.61 |
| *'F8'* | -9.50 | 2.49 | -10.57 | 2.79 | -11.39 | 2.35 | -8.29 | 1.94 | -7.07 | -0.20 | -5.34 | 3.41 |
| *'FT7'* | -5.16 | 2.45 | -6.06 | 5.75 | -5.97 | 2.82 | -6.60 | 2.35 | -6.51 | 3.36 | -5.81 | 3.08 |
| *'FC5'* | -8.22 | 6.59 | -9.70 | 9.96 | -8.23 | 7.19 | -6.06 | 4.63 | -4.95 | 4.61 | -5.22 | 3.55 |
| *'FC3'* | -4.85 | 3.12 | -3.50 | 2.29 | -5.28 | 1.68 | -7.00 | 4.09 | -6.05 | 3.41 | -4.89 | 2.90 |
| *'FC1'* | -6.66 | 4.25 | -9.33 | 2.47 | -6.06 | 1.69 | -6.50 | 3.77 | -4.97 | 2.70 | -5.29 | 4.80 |
| *'FC2'* | -8.42 | 3.73 | -10.83 | 4.89 | -7.64 | 2.54 | -6.72 | 2.99 | -4.84 | 0.87 | -5.77 | 4.95 |
| *'FC4'* | -4.82 | 5.11 | -6.27 | 4.66 | -5.31 | 6.42 | -6.51 | 2.48 | -5.47 | 1.59 | -6.11 | 3.68 |
| *'FC6'* | -5.13 | 3.46 | -5.12 | 3.80 | -5.84 | 4.69 | -4.10 | 2.05 | -4.41 | 1.96 | -5.46 | 4.21 |
| *'FT8'* | -7.46 | 3.16 | -7.71 | 3.96 | -7.80 | 6.40 | -7.30 | 4.35 | -5.11 | 1.71 | -5.32 | 2.98 |
| *'T3'* | -4.93 | 2.73 | -4.12 | 5.97 | -6.43 | 6.25 | -6.36 | 2.36 | -5.84 | 3.64 | -4.50 | 6.26 |
| *'C5'* | -4.36 | 6.03 | -7.25 | 8.20 | -7.40 | 8.26 | -6.43 | 3.33 | -5.19 | 2.57 | -5.70 | 4.58 |
| *'C3'* | -5.51 | 2.14 | -7.69 | 6.88 | -4.46 | 1.90 | -4.79 | 2.93 | -5.70 | 4.12 | -5.39 | 3.22 |
| *'C1'* | -7.09 | 8.15 | -4.31 | 2.80 | -6.08 | 2.23 | -5.64 | 4.04 | -4.68 | 2.75 | -5.06 | 3.30 |
| *'C2'* | -8.19 | 3.85 | -8.34 | 7.47 | -4.02 | 2.81 | -5.34 | 1.50 | -6.04 | 1.33 | -5.62 | 3.00 |
| *'C4'* | -5.39 | 5.19 | -6.02 | 6.69 | -7.44 | 6.35 | -5.51 | 2.01 | -5.18 | 1.96 | -5.83 | 3.81 |
| *'C6'* | -4.32 | 1.13 | -4.00 | 1.05 | -5.38 | 3.45 | -5.21 | 1.38 | -5.46 | 2.22 | -5.66 | 4.17 |
| *'T4'* | -5.22 | 4.94 | -4.36 | 6.46 | -3.15 | 6.96 | -4.08 | 2.85 | -4.54 | 2.61 | -5.12 | 3.55 |
| *'TP7'* | -6.64 | 3.90 | -6.84 | 6.89 | -3.73 | 3.35 | -4.79 | 0.54 | -5.08 | 5.03 | -5.01 | 5.59 |
| *'CP5'* | -5.53 | 7.46 | -5.88 | 4.94 | -6.02 | 4.13 | -5.52 | 2.95 | -6.30 | 3.81 | -5.64 | 4.46 |
| *'CP3'* | -5.56 | 8.00 | -3.73 | 4.45 | -3.87 | 3.71 | -5.49 | 3.42 | -5.63 | 3.81 | -5.66 | 3.82 |
| *'CP1'* | -7.25 | 6.55 | -9.67 | 6.59 | -5.10 | 4.02 | -4.38 | 1.38 | -4.62 | 1.20 | -4.90 | 2.47 |
| *'CP2'* | -7.08 | 3.59 | -6.23 | 7.52 | -6.63 | 6.67 | -6.27 | 2.78 | -5.71 | 3.04 | -5.77 | 4.03 |
| *'CP4'* | -7.64 | 5.57 | -9.47 | 9.97 | -8.47 | 9.27 | -4.34 | 1.52 | -4.85 | 2.36 | -5.64 | 4.10 |
| *'CP6'* | -5.76 | 5.14 | -7.30 | 4.23 | -5.71 | 1.60 | -5.85 | 2.33 | -4.83 | 2.93 | -5.37 | 4.72 |
| *'TP8'* | -4.58 | 4.52 | -5.43 | 7.84 | -3.35 | 6.87 | -5.35 | 3.73 | -4.10 | 2.75 | -5.67 | 5.41 |
| *'T5'* | -4.75 | 5.34 | -6.11 | 4.52 | -7.04 | 1.83 | -4.73 | 2.45 | -4.69 | 3.78 | -4.38 | 3.98 |
| *'P5'* | -7.43 | 8.15 | -8.28 | 6.97 | -6.61 | 1.75 | -4.60 | 1.60 | -5.29 | 2.63 | -4.98 | 4.04 |
| *'P3'* | -4.73 | 3.94 | -4.41 | 2.80 | -6.21 | 2.13 | -5.46 | 2.02 | -5.79 | 2.76 | -4.73 | 3.41 |
| *'P1'* | -7.04 | 4.48 | -5.34 | 1.39 | -4.75 | 5.24 | -5.15 | 1.24 | -5.12 | 2.52 | -5.03 | 3.37 |
| *'P2'* | -5.30 | 5.52 | -4.46 | 5.24 | -8.55 | 6.08 | -6.63 | 2.69 | -5.05 | 2.83 | -5.00 | 3.28 |
| *'P4'* | -5.85 | 3.93 | -3.54 | 3.18 | -8.08 | 6.55 | -9.10 | 5.91 | -4.46 | 1.75 | -5.17 | 4.10 |
| *'P6'* | -4.96 | 4.64 | -4.85 | 2.69 | -9.98 | 4.99 | -7.16 | 3.84 | -4.43 | 2.16 | -5.01 | 4.62 |
| *'T6'* | -3.32 | 4.12 | -4.00 | 2.75 | -7.40 | 3.53 | -7.47 | 4.26 | -4.51 | 2.27 | -5.14 | 4.78 |
| *'FPZ'* | -8.50 | 6.21 | -6.15 | 3.69 | -9.87 | 3.53 | -7.81 | 2.91 | -5.47 | 1.34 | -5.44 | 3.16 |
| *'PO7'* | -4.62 | 2.75 | -4.04 | 2.30 | -8.24 | 5.73 | -7.07 | 3.56 | -4.30 | 2.35 | -5.37 | 3.15 |
| *'PO3'* | -4.41 | 1.54 | -4.26 | 5.16 | -6.47 | 2.90 | -4.16 | 0.98 | -4.90 | 2.07 | -4.53 | 2.70 |
| *'O1'* | -6.87 | 4.52 | -3.87 | 1.57 | -6.69 | 2.87 | -4.55 | 1.72 | -5.05 | 2.43 | -4.17 | 2.97 |
| *'O2'* | -5.37 | 3.22 | -5.35 | 2.93 | -3.40 | 2.52 | -5.12 | 2.63 | -4.54 | 0.62 | -4.35 | 1.85 |
| *'PO4'* | -5.65 | 3.47 | -3.92 | 3.08 | -5.44 | 4.33 | -7.56 | 3.55 | -5.24 | 0.97 | -5.21 | 3.37 |
| *'PO8'* | -7.09 | 0.25 | -4.49 | 0.14 | -6.68 | 2.79 | -6.87 | 3.70 | -5.07 | 0.84 | -4.02 | 2.39 |
| *'OZ'* | -7.58 | 1.40 | -4.94 | 2.06 | -6.04 | 2.54 | -5.55 | 2.19 | -5.03 | 1.58 | -4.57 | 2.44 |
| *'AFZ'* | -7.72 | 4.20 | -5.97 | 4.18 | -9.86 | 5.81 | -7.29 | 3.12 | -5.08 | 1.02 | -5.23 | 3.10 |
| *'FZ'* | -9.72 | 6.12 | -7.44 | 4.88 | -8.51 | 7.77 | -9.18 | 2.83 | -5.40 | 0.36 | -4.54 | 3.40 |
| *'FCZ'* | -7.63 | 4.95 | -5.75 | 0.01 | -4.54 | 3.52 | -6.48 | 1.16 | -4.26 | 0.07 | -4.26 | 4.28 |
| *'CZ'* | -8.69 | 5.28 | -5.66 | 1.47 | -4.90 | 1.58 | -5.69 | 1.11 | -5.49 | 1.47 | -4.97 | 3.58 |
| *'CPZ'* | -5.03 | 4.24 | -4.61 | 3.94 | -5.58 | 6.12 | -6.25 | 1.79 | -5.45 | 2.40 | -4.75 | 3.09 |
| *'PZ'* | -5.79 | 1.82 | -4.94 | 2.19 | -6.83 | 6.80 | -7.06 | 2.13 | -5.11 | 2.09 | -5.49 | 4.49 |
| *'POZ'* | -6.28 | 4.28 | -4.76 | 3.16 | -6.87 | 6.11 | -6.32 | 2.13 | -4.84 | 1.59 | -5.12 | 3.88 |

*4.3 GE*

Confidence intervals of the GLM analyses are presented in **Supplementary Tables S11.**

**Supplementary Table S11**. Confidence interval (CI) for General Linear Model (GLM) results of Global Efficiency (GE) and Difficulty in Attunement and Engagement dimensions during the first clinical interview. The significant results (p<0.05, uncorrected) are reported in bold.

| **Domain** | **Delta** | | **Theta** | | **Alpha** | | **Beta** | | **Gamma Low** | | **Gamma High** | |
| --- | --- | --- | --- | --- | --- | --- | --- | --- | --- | --- | --- | --- |
| *Difficulty in Attunement* | -24.24 | 10.64 | -22.35 | 4.96 | -25.06 | 12.73 | -18.31 | 7.70 | -15.38 | 8.18 | -15.99 | 6.70 |
| *Engagement* | -5.14 | 3.05 | -4.94 | 3.06 | -5.04 | 2.66 | -6.25 | 2.55 | -5.02 | 1.31 | -5.03 | 2.92 |

**References**

1. Pallagrosi, M., Fonzi, L., Picardi, A. and Biondi, M. (2014) 'Assessing clinician’s subjective experience during interaction with patients', *Psychopathology*, 47(2), pp. 111–118. Available at:<https://doi.org/10.1159/000351589>.
2. Delorme, A. and Makeig, S. (2004) 'EEGLAB: An open source toolbox for analysis of single-trial EEG dynamics including independent component analysis', *Journal of Neuroscience Methods*, 134(1), pp. 9–21. Available at:<https://doi.org/10.1016/j.jneumeth.2003.10.009>.
